# Polyamine-Induced Hormonal Changes in *eds5* and *sid2* Mutant *Arabidopsis* Plants

**DOI:** 10.3390/ijms20225746

**Published:** 2019-11-15

**Authors:** Judit Tajti, Kamirán Áron Hamow, Imre Majláth, Krisztián Gierczik, Edit Németh, Tibor Janda, Magda Pál

**Affiliations:** 1 Plant Physiology Department, Agricultural Institute, Centre for Agricultural Research, H-2462 Martonvásár, Hungary; tajti.judit@agrar.mta.hu (J.T.); majlath.imre@agrar.mta.hu (I.M.); nedokka@gmail.com (E.N.); janda.tibor@agrar.mta.hu (T.J.); 2 Plant Protection Institute, Centre for Agricultural Research, H-2462 Martonvásár, Hungary; hamow.kamiran@agrar.mta.hu; 3 Department of Plant Molecular Biology, Agricultural Institute, Centre for Agricultural Research, H-2462 Mrtonvásár, Hungary; gierczik@gmail.com

**Keywords:** polyamine, salicylic acid, plant hormone, *Arabidopsis*, *eds5-1*, *sid2-2*

## Abstract

Polyamines are multifaceted compounds which play a role in regulating plant growth and stress tolerance in interactions with plant hormones. The aim of the present study was to reveal how exogenous polyamines influence the synthesis of salicylic acid, with a special emphasis on the effect of salicylic acid deficiency on the polyamine metabolism and polyamine-induced changes in other plant hormone contents. Our hypothesis was that the individual polyamines induced different changes in the polyamine and salicylic acid metabolism of the wild type and salicylic acid-deficient *Arabidopsis* mutants, which in turn influenced other hormones. To our knowledge, such a side-by-side comparison of the influence of *eds5-1* and *sid2-2* mutations on polyamines has not been reported yet. To achieve our goals, wild and mutant genotypes were tested after putrescine, spermidine or spermine treatments. Polyamine and plant hormone metabolism was investigated at metabolite and gene expression levels. Individual polyamines induced different changes in the *Arabidopsis* plants, and the responses were also genotype-dependent. Polyamines upregulated the polyamine synthesis and catabolism, and remarkable changes in hormone synthesis were found especially after spermidine or spermine treatments. The *sid2-2* mutant showed pronounced differences compared to Col-0. Interactions between plant hormones may also be responsible for the observed differences.

## 1. Introduction

Polyamines (PAs) are low molecular weight aliphatic amines containing two or more amino groups found in all living cells [1]. In higher plants, PAs are mainly present in their free form, and have several potent biological activities. Thus, PAs are considered to be a new group of growth regulators in plants [2]. The total and individual PAs levels vary depending on the plant species, the organ and on the developmental stage; furthermore, their metabolism is dynamic, due to the polyamine cycle [3]. Experiments on treatments with both PAs and PA synthesis inhibitors revealed the role of PAs in e.g., flower induction [4], embryogenesis [5], regulation of nucleic acid synthesis and protein translation, development of organelles [6], and senescence [7], and numerous studies have been published on the relationship between the enhanced synthesis of PAs and the level of a stress tolerance [8,9,10]. A positive role of the early stress-induced activation or the overexpressing of certain PA-biosynthesis genes has also been proved (arginine decarboxylase: *ADC*, spermidine synthase: *SPDS* and spermine synthase: *SPMS*) [11,12,13,14,15,16]. In addition, microarray studies have shown that increased endogenous PA content can alter the gene expression level of genes involved in the synthesis and signalling of several plant hormones, such as abscisic acid (ABA), auxin, ethylene, gibberellins (GAs), jasmonic acid (JA) or salicylic acid (SA) in transgenic *Arabidopsis* plants [17]. It is thus becoming more evident that PAs are also switching points in plant signalling pathways, and the induced plant responses are interconnected at many levels with other metabolic routes and hormonal cross-talk and activate gene expression, which has a predominant role in the PA-signalling processes compared only to PA accumulation [18].

Most studies have focused on the protective effects of exogenous PAs, but their role is more complicated. It is difficult to establish a direct relationship between PAs, especially the individual levels of the most abundant Pas—namely putrescine (PUT), spermidine (SPD) and spermine (SPM)—and the observed beneficial effects. In addition, an excess of PAs can be harmful to the plants [15]. According to these facts, changes in the PA levels are important for fine-tuning PA signalling, which influences the hormonal balance required for them to exert their positive role in regulating plant growth and stress tolerance [15].

Earlier it was demonstrated that SA treatment affects the synthesis and/or catabolism of PAs [19,20,21,22]. Vice versa, it was shown that SPD treatment increased SA content in the leaves of wheat, but PUT or SPD treatments decreased it in the roots [23], whereas SPD did not affect endogenous SA content in cucumber [24]. In our previous study, it was found that there is a close, positive relationship between PAs and SA accumulation after SPD and SPM treatments in wheat and maize plants [15]. SA synthesis starts from chorismate as a precursor, after which the synthesis pathway branches into two separate pathways. SA can be synthesized through the phenylalanine ammonia-lyase (PAL) pathway from phenylalanine via benzoic acid (BA), while on the isochorismate pathway the enzyme isochorismate synthsase (ICS) catalyzes isochorismate formation from chorismate. In *Arabidopsis*, the primary route for SA production is the isochorismate pathway, and there are two genes *ICS1* and *ICS2* encoding ICS, but it was demonstrated that ICS1 is responsible for the main source of isochorismate [25]. Excessive SA accumulation can be detrimental to plants under stress conditions. For example, a negative relationship was observed between the SA content and growth parameters of *cpr1 Arabidopsis* mutant plants, which showed higher levels of free and bound SA and increased oxidative damage under low temperature stress conditions [26]. Increased SA accumulation was also responsible for the negative effect of SPD and SPM treatments, especially at higher concentration, manifested in growth inhibition in wheat and maize under control conditions [15], and also for the accelerating effect of SPD treatment on cadmium-induced oxidative stress in wheat [16].

However, during the investigation on the relationship between SA and PAs, it should be also taken into consideration that SA may also influence other plant hormones, e.g SA has an antagonistic relation with ABA and JA [27,28], thus the PA-induced hormonal changes may also be affected.

A positive feedback loop has also been found between ABA and PAs, as ABA activates the PA synthesis genes and also that of polyamine oxidase (*PAO*); moreover PA treatment induces ABA synthesis at the gene expression level of 9-*cis*-epoxycarotenoid dioxygenase (*NCED)* [29,30,31]. In a recent study, PA treatments differently influenced the plant growth parameters of GA-insensitive dwarf (carrying the severe dwarfing allele *Rht-B1c*, responsible for dwarf phenotype) and semi-dwarf Rht (carrying *Rht-B1b* allele) wheat lines compared to the wild, tall line, which effects were in relation with different changes in ABA and SA contents of the three genotypes [32]. These results provide new insights into the role of PAs in plant growth regulation and confirmed their participation in the hormonal balance, however, still little is known about the interplay between PAs and GAs. Methyl jasmonate treatment in barley induced the expression of genes involved in PUT synthesis *ADC* and ornithine decarboxylase, which in turn led to increased PA content in the conjugated fraction [33]. *OsPAO6* has been also reported to be induced by JA [34]. Exogenous PAs, especially SPM, induced JA accumulation after only 1 h of treatment [35].

In order to reveal even more deeply the relationship between PAs and SA at the metabolite and gene expression levels, and the contribution of SA synthesis deficiency to the interplay of PA with other plant hormones, such as ABA, GAs and JA, in the present study, *eds5* and *sid2* mutants of *Arabidopsis*—which do not accumulate SA after pathogen infection or abiotic stresses [36,37]—were tested after PUT, SPD or SPM treatments. Our hypothesis was that the different Pas would induce different changes in the PA and SA metabolism of the wild type and SA-deficient *Arabidopsis* mutants, which in turn would influence other plant hormones. To our knowledge, such a side-by-side comparison of the influence of *eds5* and *sid2* mutation on the effect of different PAs (PUT, SPD and SPM) has not been reported yet. The main questions were: (1) Is there any difference in the PA metabolism between the SA-deficient mutants and the wild type? (2) How do exogenous PAs influence SA synthesis? and (3) Does SA-deficiency have any influence on the PA-induced changes in the ABA, JA and GA contents?

## 2. Results

### 2.1. Treatments with PA Repressed the Effective Quantum Yield of PSII

**Figure 1:**
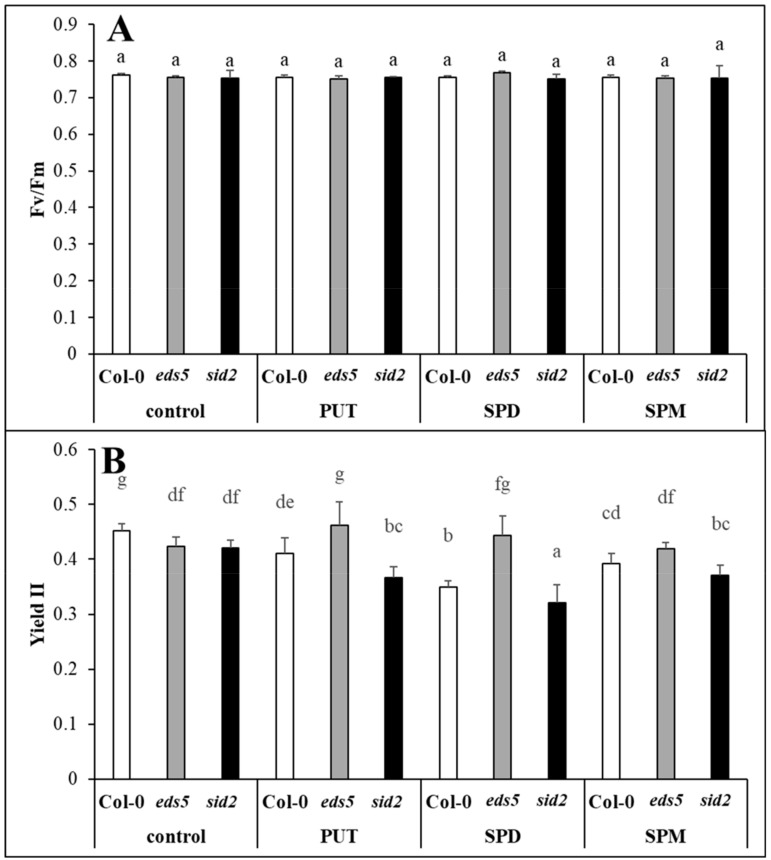
Effect of 0.5 mM 1-day of putrescine (PUT), spermidine (SPD) and spermine (SPM) treatments on the chlorophyll-*a* fluorescence induction parameters ((**A**) Fv/Fm: maximum quantum yield of PSII photochemistry, (**B**) YII: Effective PSII quantum yield) in Col-0, wild type, *eds5-1* (*eds5*) and *sid2-2* (*sid2*) *Arabidopsis* mutants. Data represent mean values ± SD. Different letters indicate significant differences at *p* ≤ 0.05 level.

In order to get information about the physiological status of the plants, certain Chl-*a* fluorescence parameters were determined in Col-0, *sid2* and *eds5* plants treated with or without different PAs. The Fv/Fm chlorophyll-*a* fluorescence induction parameter representing the maximum quantum efficiency of PSII, showed similar values in the three genotypes, and was not influenced by any of the PA treatments, indicating that exogenous PA application did not induce severe stress conditions in *Arabidopsis* plants (Figure 1A). However, the effective PSII quantum yield (YII) was significantly lower in the two mutants compared to the wild type, and decreased by all the applied PAs in Col-0 and *sid2* genotypes, while it slightly increased in PUT-treated *eds5* mutant (Figure 1B, Figure S1).

### 2.2. Effect of Exogenous PAs on PA Contents and Metabolism

#### 2.2.1. Exogenous PAs Triggered Endogenous PUT Accumulation by Promoting ADC2 Transcription

Under control conditions, no significant differences were observed between the mutants and the Col-0, wild type regarding on the PUT content or the expression levels of its synthesis genes (*ADC1* and *ADC2*). 

**Figure 2:**
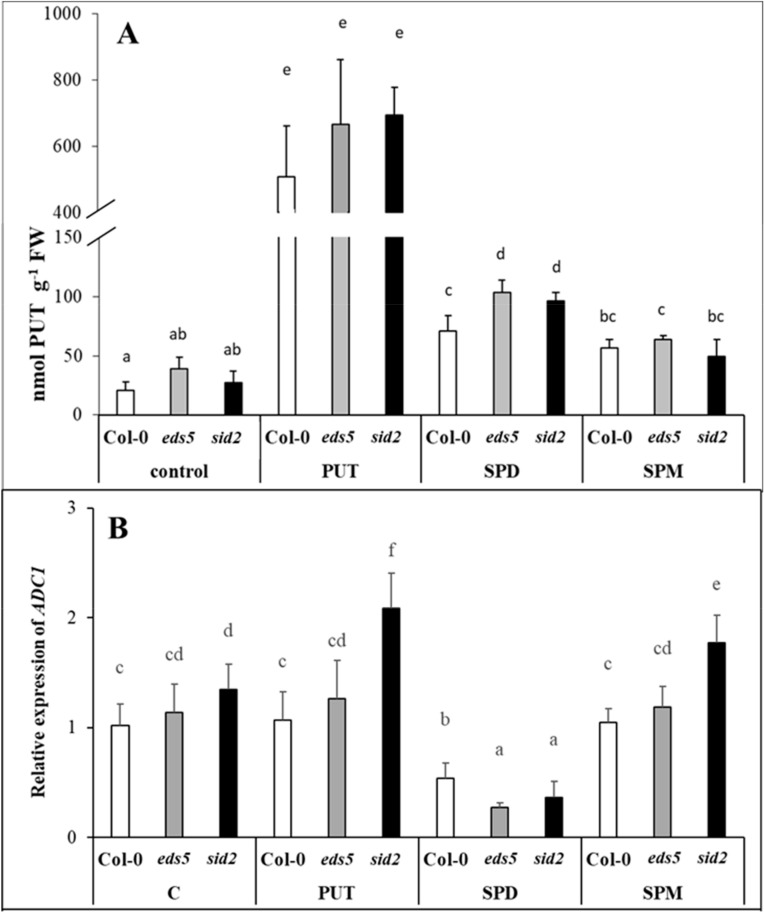

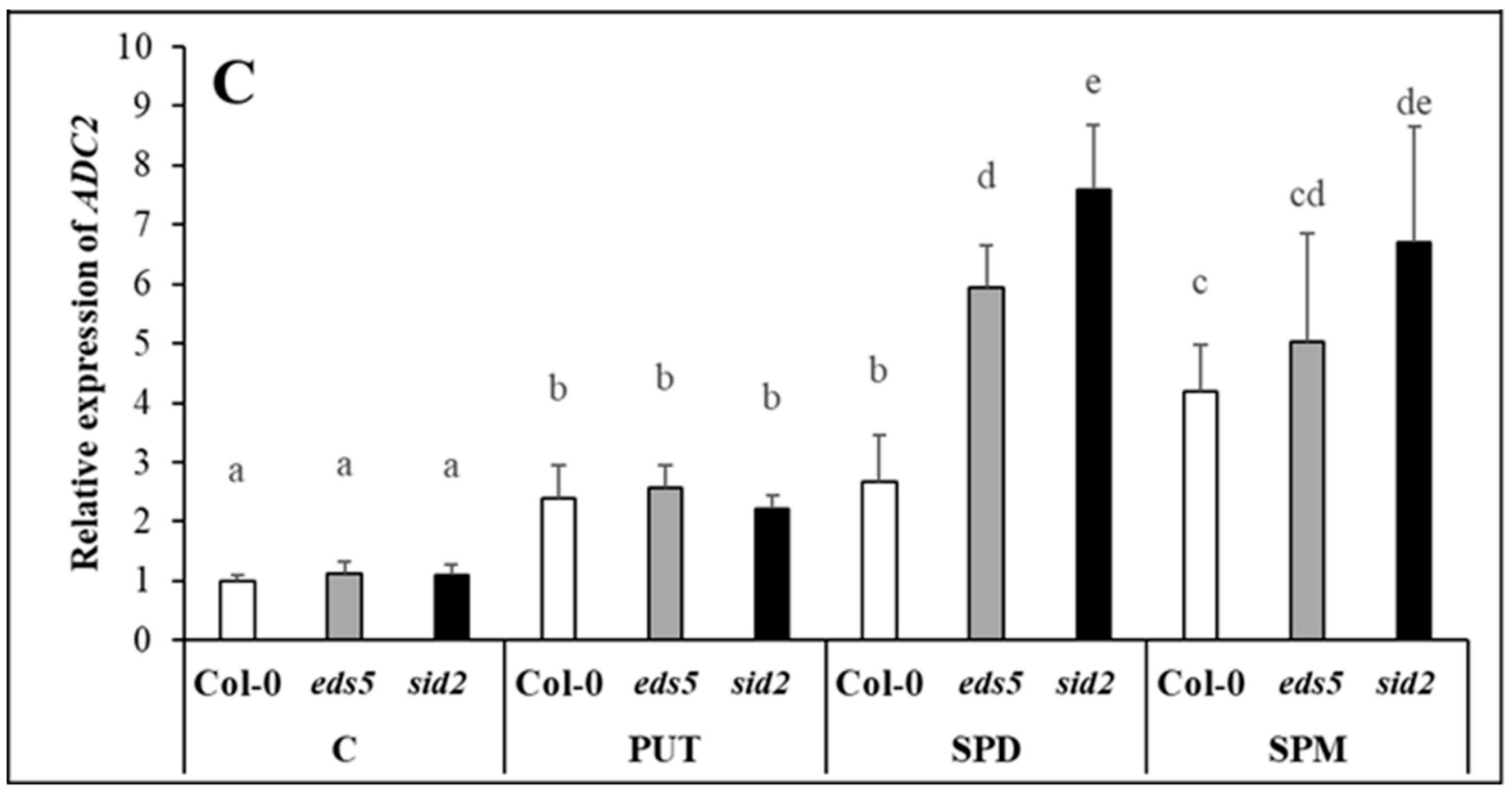
Effect of 0.5 mM 1-day of putrescine (PUT), spermidine (SPD) and spermine (SPM) treatments on the endogenous putrescine content ((**A**) PUT) and on the expression levels of putrescine synthesis genes (**B**,**C**) *ADC1-2: arginine decarboxylase1-2*) in Col-0, wild type, *eds5-1* (*eds5*) and *sid2-2* (*sid2*) *Arabidopsis* mutants. Data represent mean values ± SD. Different letters indicate significant differences at *p* ≤ 0.05 level.

After PA treatments, pronounced changes were observed in the PUT contents of *Arabidopsis* leaves, as all the applied treatments increased them, with the highest accumulation being in the case of PUT treatment, followed by SPD and SPM treatments (Figure 2A, Figure S2A). The expression patterns of *ADC1* and *ADC2* showed that PA treatments differentially regulated them (Figure 2B,C, Figure S2B,C). *ADC1* was only slightly induced in PUT- and SPM-treated *sid2* and down-regulated after SPD treatment in all the investigated genotypes, but *ADC2* was significantly induced by all the PA treatments both in the wild type and the mutants. Although the basal gene expression level of *ADC2* did not differ between the three genotypes, interestingly, both after SPD and SPM treatments the highest expression level of *ADC2* was observed in the *sid2* mutant (Figure 2C).

#### 2.2.2. Treatment with Higher PAs Down-Regulated the Expression of Genes Involved in SPD Synthesis

**Figure 3:**
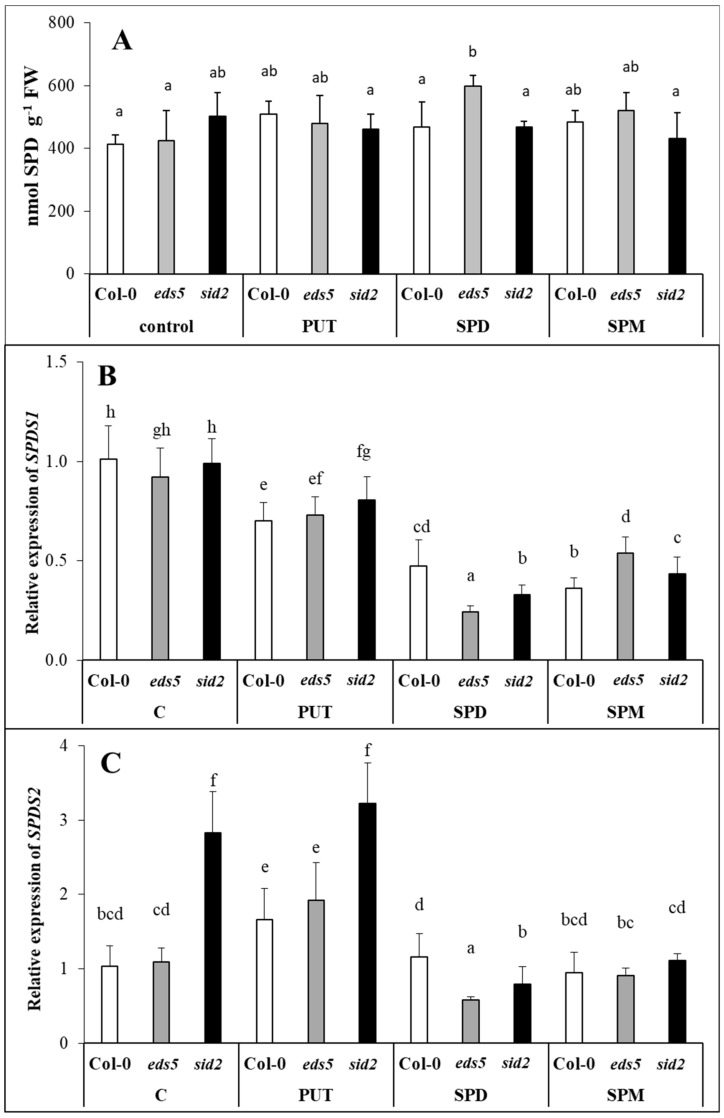
Effect of 0.5 mM 1-day of putrescine (PUT), spermidine (SPD) and spermine (SPM) treatments on spermidine content ((**A**) SPD) and on the expression levels of spermidine synthesis genes (**B**,**C**) *SPDS1-2*: spermidine synthase1-2) in Col-0, wild type, *eds5-1* (*eds5*) and *sid2-2* (*sid2*) *Arabidopsis* mutants. Data represent mean values ± SD. Different letters indicate significant differences at *p* ≤ 0.05 level.

The initial SPD content of the Col-0, *eds5* or *sid2* genotypes did not differ (Figure 3A, Figure S3A), and the expression level of *SPDS1* was also similar (Figure 3B, Figure S3B), but a definite difference was found in the level of *SPDS2* transcript between the Col-0/*eds5* and *sid2* genotypes under control conditions (Figure 3C, Figure S3C), as was almost three-fold higher in the latter one. Although the SPD content was not influenced pronouncedly, the gene expression pattern significantly changed after exogenous PA treatments. While PUT treatment decreased the expression of *SPDS1* in either of the genotypes, rather increased that of *SPDS2* in the case of Col-0 and *eds5*. After SPD or SPM treatments the *SPDS1* expression was down-regulated in both the wild and mutant plants, while the initial differences in *SPDS2* expression observed under control conditions were disappeared, as decreased in the *sid2* mutant to a similar level as it was observed for Col-0 (Figure 3B,C). The decrease in *SPDS2* transcript after SPD or SPM treatments was more observable compared to the PUT-treated ones.

#### 2.2.3. Exogenous PAs Up-Regulated the Expression of SPDS

**Figure 4:**
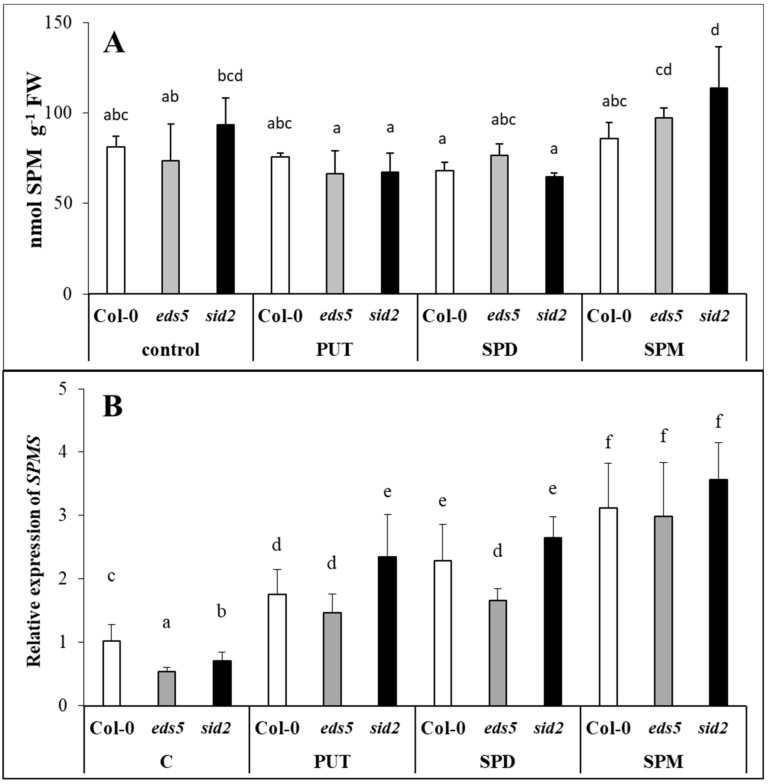
Effect of 0.5 mM 1-day of putrescine (PUT), spermidine (SPD) and spermine (SPM) treatments on spermine content ((**A**) SPM) and on the expression levels of spermine synthase gene ((**B**) *SPMS*) in Col-0, wild type, *eds5-1* (*eds5*) and *sid2-2* (*sid2*) *Arabidopsis* mutants. Data represent mean values ± SD. Different letters indicate significant differences at *p* ≤ 0.05 level.

The SPM content in the control plants were in the same range. Significant changes were not detected after PA treatments, except for *sid2*, where SPM treatment increased the SPM content, if it was compared only to the PUT- or SPD-treated ones (Figure 4A, Figure S4A). Despite to these, exogenous application of PUT, SPD or SPM significantly induced the SPMS expression in either of the genotypes, regardless of the type of the mutation, with highest increment in case of the SPM treatment (Figure 4B, Figure S4B).

#### 2.2.4. Exogenous PAs Induced the Terminal Catabolism and Not the Back-Conversion of the PAs

**Figure 5:**
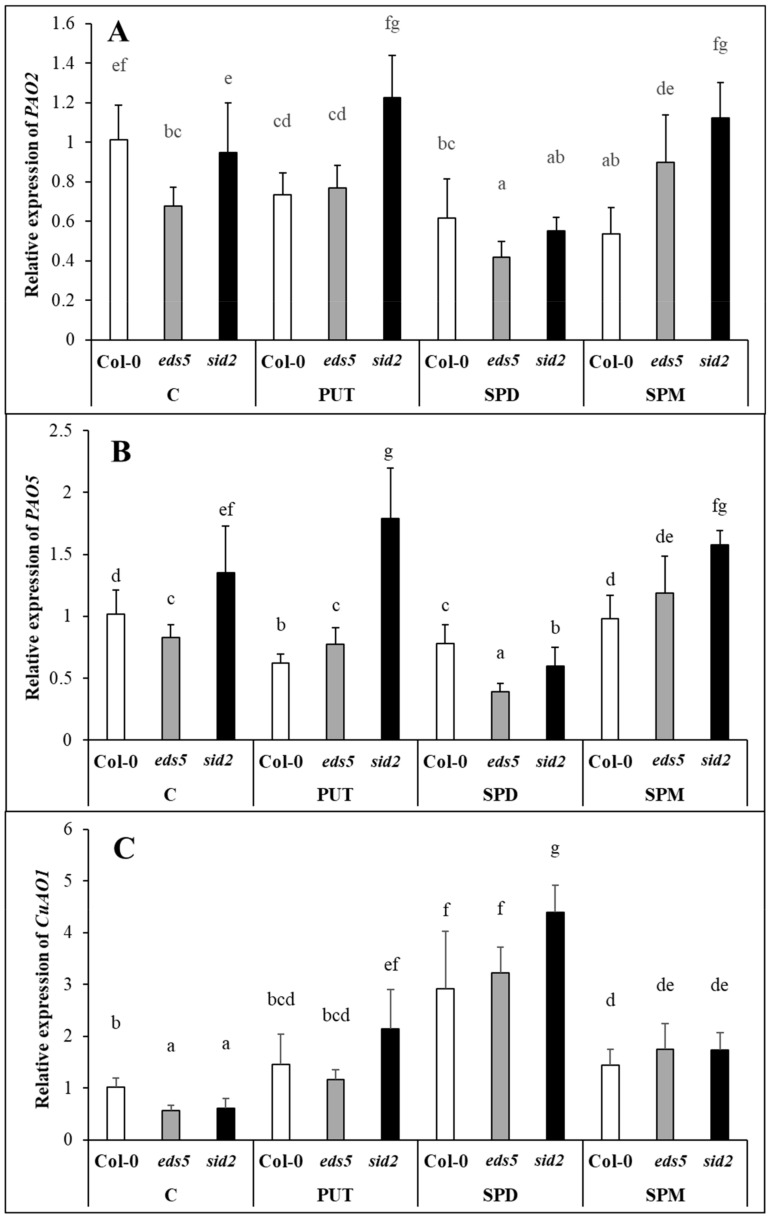
Effect of 0.5 mM 1-day of putrescine (PUT), spermidine (SPD) and spermine (SPM) treatments on the expression levels of polyamine metabolism genes (**A**,**B**) *PAO2-5*: polyamine oxidase2-5, (**C**) *CuAO1*: cooper amine-oxidase1) in Col-0, wild type, *eds5-1* (*eds5*) and *sid2-2* (*sid2*) *Arabidopsis* mutants. Data represent mean values ± SD. Different letters indicate significant differences at *p* ≤ 0.05 level.

The expression levels of the genes encoding PAOs, which are responsible for the back-conversion of the higher polyamines, namely *PAO2* and *PAO5*, and that of *CuAO1*, encoding the copper amine oxidase responsible for terminal catabolism of PUT and SPD, were similar in the three genotypes. *PAO2* and *PAO5* transcript levels did not show remarkable changes after PUT or SPM treatments, but were slightly decreased by SPD in all the genotypes (Figure 5A,B, Figure S4A,B), while the expression of *CuAO1* was induced after all the PA treatments, especially after SPD application (Figure 5C, Figure S5C). An opposite pattern of the expression of *PAOs* and *CuAO1* was seen after SPD treatment, indicating that it rather induced the catabolism and not the conversion back to PUT. Interestingly, the *sid2* mutant showed the highest value in several cases.

### 2.3. Exogenous PAs Induced the PAL Synthesis Pathway

**Figure 6:**
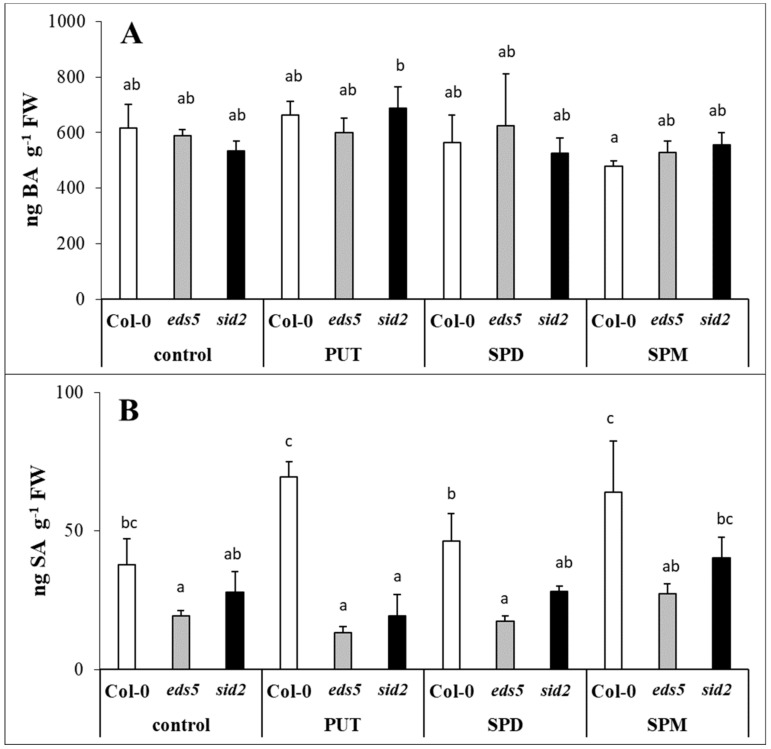
Effect of 0.5 mM 1-day of putrescine (PUT), spermidine (SPD) and spermine (SPM) treatments on the contents of benzoic acid ((**A**) BA) and salicylic acid ((**B**) SA) in Col-0, wild type, *eds5-1* (*eds5*) and *sid2-2* (*sid2*) *Arabidopsis* mutants. Data represent mean values ± SD. Different letters indicate significant differences at *p* ≤ 0.05 level.

The BA contents of the Col-0 and *eds5* genotypes were almost similar under control conditions and PA treatments did not substantially influenced it (Figure 6A, Figure S6A). Although, as it was expected, pronounced differences were observed in the SA content of the control plants, as its level was lower in the two mutant genotypes, and these differences still remained after the PA treatments (Figure 6B, Figure S6B), exogenous PAs could hardly induce significant changes in the SA contents.

**Figure 7:**
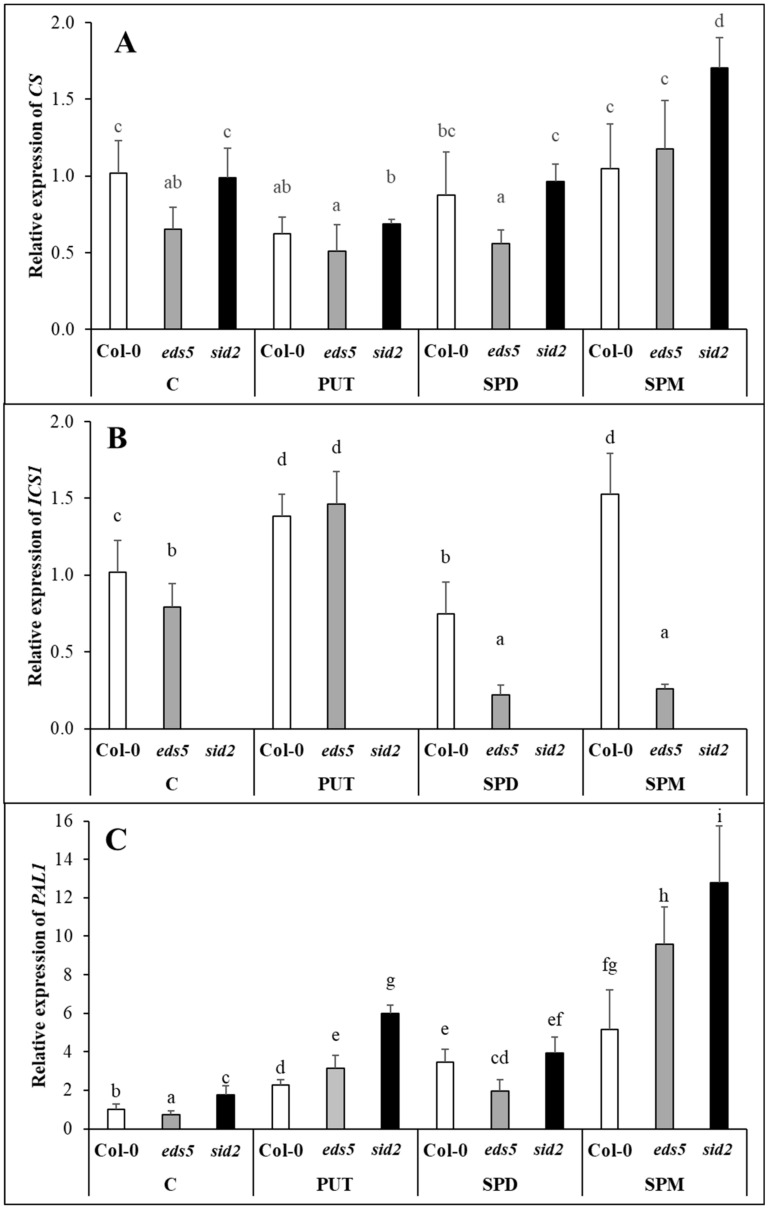
Effect of 0.5 mM 1-day of putrescine (PUT), spermidine (SPD) and spermine (SPM) treatments on the expression levels of genes involved in salicylic acid synthesis ((**A**) *CS*: chorismate synthase, (**B**) *ICS1*: isochorismate synhtase1, (**C**) *PAL1*: phenylalanine ammonia-lyase1) in Col-0, wild type, *eds5-1* (*eds5*) and *sid2-2* (*sid2*) *Arabidopsis* mutants. Data represent mean values ± SD. Different letters indicate significant differences at *p* ≤ 0.05 level.

The initial differences in the expression levels of genes involved in SA synthesis were not substantial, except for that *ICS1* was not expressed in the *sid2* mutant. The gene expression level of *CS* was slightly down-regulated in PUT-treated Col-0 and *sid2* plants, but compared to this, it increased in SPM-treated *sid2* plants (Figure 7A, Figure S7A). While the expression of *ICS1* was induced in PUT-treated *eds5*, it decreased after SPD and SPM treatments (Figure 7B, Figure S7B). Interestingly, in the wild type *Arabidopsis* plants the level of SA showed similar pattern to the transcript level of *ICS1*. Compared to these, the *PAL1* expression, likely as a compensation of the mutation of the *ICS1*, showed higher basal level in *sid2* plants (Figure 7C, Figure S7C). In addition, all the PA treatments induced it, with the highest levels in *sid2* mutant, and in case of the SPM treatment.

### 2.4. Exogenous PAs Induced ABA Synthesis

**Figure 8:**
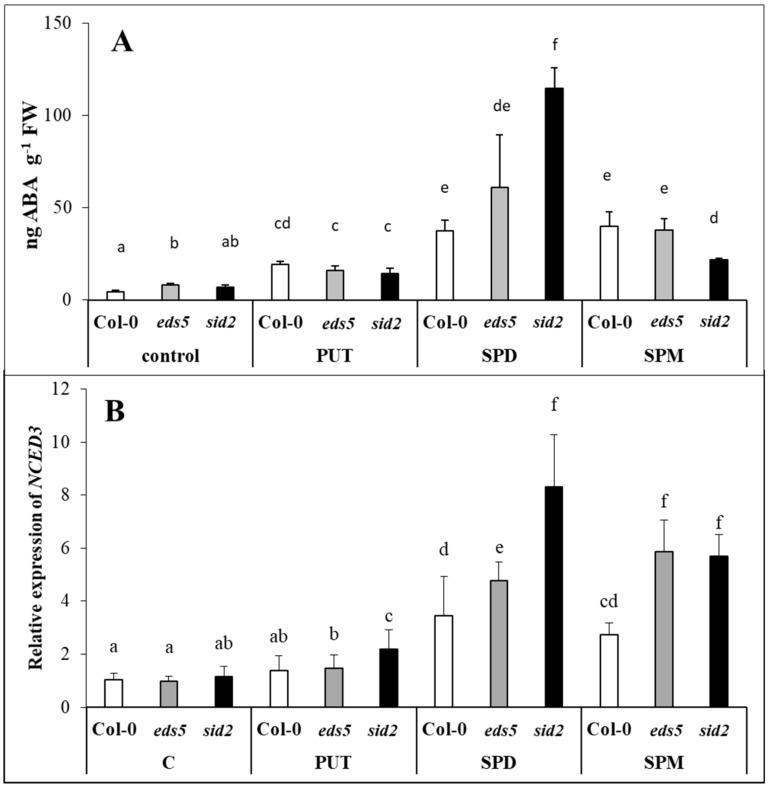
Effect of 0.5 mM 1-day of putrescine (PUT), spermidine (SPD) and spermine (SPM) treatments on the content of abscisic acid ((**A**) ABA) and the expression level of gene involved in abscisic acid synthesis ((**B**) *NCED3:* 9-cis-epoxycarotenoid dioxygenase3) in Col-0, wild type, *eds5-1* (*eds5*) and *sid2-2* (*sid2*) *Arabidopsis* mutants. Data represent mean values ± SD. Different letters indicate significant differences at *p* ≤ 0.05 level.

The three genotypes had a similar initial ABA content. All the PA treatments increased the ABA level, interestingly with the highest accumulation in SPD-treated *sid2* mutant (Figure 8A, Figure S8A), where a pronounced difference was observed between the wild types and the *sid2* mutant. The final plastid-localized steps in ABA synthesis is catalysed by NCED. The expression level of *NCED* (Figure 8B, Figure S8B) was in accordance with the changes in ABA content, as the PA treatments up-regulated it, with the highest level in SPD-treated *sid2* mutant.

### 2.5. Exogenous PAs Differently Influenced JA Content and AOS Expression

**Figure 9:**
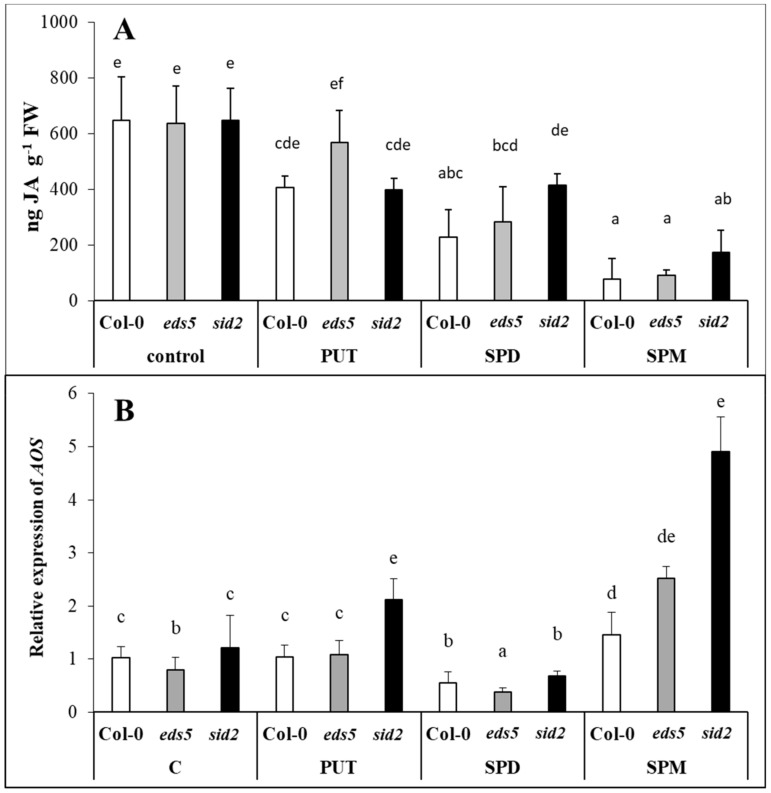
Effect of 0.5 mM 1-day of putrescine (PUT), spermidine (SPD) and spermine (SPM) treatments on the content of jasmonic acid ((**A**) JA) and the expression level of gene involved in jasmonic acid synthesis ((**B**) *AOS:* allene oxide synthase) in Col-0, wild type, *eds5-1* (*eds5*) and *sid2-2* (*sid2*) *Arabidopsis* mutants. Data represent mean values ± SD. Different letters indicate significant differences at *p* ≤ 0.05 level.

The JA content did not show any differences between the three genotypes under control conditions or after PA treatments (Figure 9A, Figure S9A). However, a decreasing trend was observed after all the PA treatments, with the highest degree in the case of the SPM treatment. The expression level of the gene encoding allene oxide synthase (AOS), which is one of the synthesis enzymes of JA biosynthesis (Figure 9B, Figure S9B), showed an opposite trend to the changes observed in JA content, as a remarkable induction of it was found in the SPM-treated plants, where the lowest JA accumulation was detected. In addition, remarkable difference was observed in the *AOS* expression of the SPM-treated wild type and *sid2* mutant.

### 2.6. PUT and SPD Induced, While SPM Down-Regulated the Expression of GA3ox1

**Figure 10:**
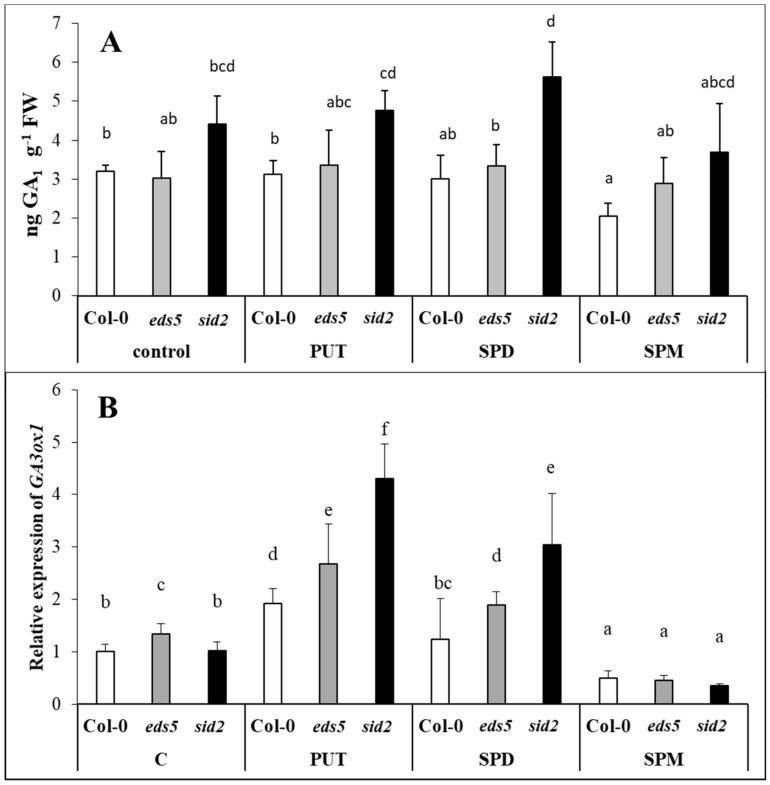
Effect of 0.5 mM 1-day of putrescine (PUT), spermidine (SPD) and spermine (SPM) treatments on the content of gibberellic acid ((**A**) GA1) and the expression level of gene involved in gibberellic acid ((**B**) *GA3ox1:* gibberellin 3-oxidase) in Col-0, wild type, *eds5-1* (*eds5*) and *sid2-2* (*sid2*) *Arabidopsis* mutants. Data represent mean values ± SD. Different letters indicate significant differences at *p* ≤ 0.05 level.

The major bioactive GAs in plants are GA_1_ and GA_4_; and the final step of the synthesis of these bioactive GAs is catalysed by gibberellin 3-oxidase (GA3ox). Among the monitored GA_1, 4, 8, 3, 20_, only the GA_1_ could be detected. Although under our experimental conditions no significant difference was detected between the three genotypes, and the PA treatments did not induce remarkable changes in the GA_1_ content, a trend towards a higher level of GA_1_ could be detected in the *sid2* mutant *Arabidopsis* (Figure 10A, Figure S10A). Interestingly, the *GA3ox1* expression level increased after PUT treatment, and slightly increased also after SPD, but decreased after SPM treatment. In PUT- and SPD-treated plants, the highest up-regulation was found again in *sid2* mutants (Figure 10B, Figure S10B).

## 3. Discussion

The main aims of the present study were to reveal the possible effects of the *eds5* and *sid2* mutations on the polyamine metabolism in *Arabidopsis* plants, and to evaluate the effects of exogenous PA on SA synthesis, and the influence of SA-deficiency on the PA-induced hormone synthesis.

Besides the visually observed phenotypical differences between the two SA-deficient mutants and wild type, the Y(II) parameter also showed that the two mutants have lower PS II quantum efficiency compared to the Col-0. Similarly, although no differences were observed in the Fv/Fm, but slightly lower effective PSII quantum yields were measured for the SA-deficient *sid2* mutant and *NahG* transgenic line compared to the wild type [38]. Parallel with these, slightly lower stomatal conductance and CO_2_ assimilation rate, and lower biomass parameters were measured for *sid2* mutant. These results confirmed that controlled levels of SA are required for optimal photosynthesis. However, SA deficient mutants, namely *eds5* and *sid2* have not been tested under PA treatments yet. Under the present conditions, PA treatments repressed the efficiency of PSII, based on the Y(II) parameter, especially in Col-0 and *sid2* genotypes, while a slight positive effect of PUT treatment has been detected in the case of *eds5* mutant compared the its control. PUT treatment at the same concentration has been reported to have a corroborating effect under control conditions in wheat plants, manifested in higher shoot fresh and dry weight and CO_2_ assimilation rate, and the beneficial effect of PUT was accompanied with the lowest SA accumulation [15]. In the present study, PAs induced the lowest decrease in Y(II) in the case of *eds5*, where interestingly the lowest SA values were detected.

Under control conditions, the *eds5* and *sid2* mutations did not influence the PA contents, among the genes involved in PUT, SPD and SPM synthesis, back-conversion or terminal catabolism, only the expression levels of SPMS showed higher transcript level in *sid2* compared to the *eds5* and the wild type. PA treatments induced remarkable increments only in the PUT content, but these were similar in the three genotypes, with the highest values in the case of PUT-treated plants. Besides the PUT uptake and translocation into the leaves, which was predominant in PUT-treated plants, *de novo* PUT synthesis was also occurred, as the expression level of *ADC2* was significantly induced by all the PA treatments, especially after SPD and SPM treatments. Our results regarding the different inducibility of the expression of *ADC1* and *ADC2*, are in accordance with the literature, as it seems that *AtADC1* is constitutively expressed, while *AtADC2* is responsive to abiotic stresses, plant hormones or pathogens [39]. Interestingly, under the present conditions, the initial transcript level of *ADC1* was slightly higher in *sid2*, and this difference became more pronounced after PUT and SPM treatment, while *ADC2* expression level was higher in *sid2* mutants after SPD or SPM treatments compared to the wild type. The *PAO2* and *PAO5* expressions in the present experiment showed exactly the same pattern, and did not show remarkable changes, except for in SPD-treated plants, where they were down-regulated. As *AtPAO2* and *AtPAO5* encoding polyamine oxidases [40,41], which are responsible for the back-conversion of higher PAs to PUT, it is not surprising that their expression was not induced upon increased PUT content and there was no need for back-conversion [42]. Except for SPD treatment, the expression level of the *PAO*s, similarly to the genes of PUT synthesis, was the highest in the *sid2* mutant. At the same time, the expression of *CuAO1*, that encoding an amine oxidase catalyses the terminal catabolism of PUT and SPD, increased after all the PA treatments, especially in case of SPD, the most pronouncedly in the *sid2* genotypes. Although, the activation of terminal catabolism was not sufficient to compensate the increased PUT content, and the differences in its pattern were not manifested in the PUT contents.

Parallel with these, although the level of higher PAs did not change in the leaves of the plants, the *SPDS1* expression level decreased after SPD or SPM treatments, suggesting that higher PAs, uptaken by the roots is probably not transported to the leaves, inducing the down-regulation of the SPD synthesis in the leaves. A decrease in *SPDS1* expression together with *CuAO1* expression in the SPD- and SPM-treated plants may be responsible for the maintenance of optimal SPD content. It has been reported that significantly induced expression of *CuAO1* was observed after ABA treatment in *Arabidopsis* [43]. In the present experiment, the highest *CuAO1* transcript level was detected parallel with the highest ABA content in the SPD-treated plants. Interestingly, under control conditions and after PUT treatment, the *SPDS2* transcript level was higher in the *sid2* mutant than in the wild type, but this difference disappeared in SPD- or SPM-treated plants, as the application of higher amounts of PAs inhibited its expression in this mutant. The up-regulation of *SPMS* was observed after all the PA treatments, which is understandable in PUT- and SPD-treated plants, where in order to decrease the uptaken excess of PUT or SPD, further synthesis was needed to SPM. Despite of these, the SPM content did not show significant increment in either of the SPM-treated plants after 1 day of treatment.

In the present experiment, the SA level was lower in the *eds5* and *sid2* mutants compared to the wild type. As *EDS5* encodes a membrane protein, located at the chloroplast envelope and responsible for SA transport [44,45], in the *eds5* mutant, the accumulated SA is trapped in the chloroplast, which in turn can inhibit *ICS* expression. While in *sid2* mutants, the SA accumulation can be only a fraction of that of the wild type, because of the lack of the ICS enzyme/pathway. Different concentrations of SA have been reported to have different effects on PA metabolism [21,46]. In addition, PA treatments, both as seed soaking or applied hydroponically efficiently enhanced SA content in wheat or maize [15,16,47]. Under the present conditions, PUT and SPM treatments after 1 day could only cause a slight, but statistically not significant increase in SA content of Col-0 genotype. Although, *CS* expression did not change after either of the PA treatments, the *ICS* transcript level in Col-0 was in correlation with the changes in SA content, as it increased after PUT or SPM treatments. Interestingly, PUT also increased the *ICS* expression in the *eds5* mutant, but SPD and SPM treatments decreased it. The latter was probably due to the inhibitory feedback effect of the SA accumulation in the chloroplasts, which was not manifested in statistically significant increase in the total SA content. The *PAL1* expression, maybe in order to compensate the mutation of the *ICS1*, showed slightly higher basal level in *sid2* plants. Although, PA treatments induced *PAL1* expression in all the genotypes and its up-regulation was the highest in SPM-treated plants, in case of all the treatments it was the highest in the *sid2* mutant. The activity of PAL, a crucial enzyme in the synthesis of flavonoids, anthocyanins and simple phenolic acids, increased after PUT treatment in the leaves of maize [15]. In addition, in *Atpao4* plants with increased SPM content, genes involved in flavonoid and/or lignin biosynthesis, such as *PAL1* were induced [48], suggesting that the accumulation of PAs increased the synthesis of phenolic compounds. According to the present results, PA treatments differently induced the SA synthesis pathways. In the Col-0 both pathways activated, especially in PUT- and SPM-treated ones. In the *eds5* mutant, after PUT treatment both pathways induced, and after SPD and SPM treatments the expression level of *PAL* further increased, but that of *ICS1* decreased. In the case of *sid2* mutant, the drastic increment in *PAL1* transcript level will be responsible for SA synthesis.

PUT and ABA are integrated in a positive feedback loop [18]. Modulation of PA metabolism at transcriptional level by ABA has been proved in *Arabidopsis* in case of PA biosynthesis genes, such as *ADC2*, *SPDS1* and *SPMS* [49]. The transcriptional regulation *NCED* in PA-overproducer plants has been demonstrated [17,50,51]. Conversely, the suppression of *ADC* resulted in the reduced expression of *NCED* and the down-regulation of ABA-regulated genes [30]. In the present experiment, PAs induced ABA accumulation and the up-regulation of *NCED*, which was the most pronounced in SPD-treated plants, where it was the highest in *sid2* mutant. This can be resulted from the well-known antagonistic relationship between SA and ABA [27,52,53]. ABA has been also reported to influence the catabolism of PAs, as its exogenous application induced the expression of *CuAO1* in *Arabidopsis* [42,43], and *PAO* in *Medicago sativa* [54] and wheat [29]. Interestingly, under the present conditions, the highest *CuAO1* expression was detected in the SPD-treated plants, where the highest ABA accumulation and *NCED* expression was found. Nevertheless, all these parameters were the highest in the *sid2* mutant plants. Similarly, higher ABA content has been also detected in *sid2 Arabidopsis* mutant compared to the wild type during the transition from pre-reproductive to reproductive stages [55], confirming that SA content may influence, at least to some extent, the endogenous concentrations of ABA.

Similar JA content was detected in *sid2* and Col-0 genotypes despite of the different SA level under control conditions and after infection with *Pythium irregular*, indicating that SA did not inhibit the JA accumulation [55,56]. In the present experiment, different SA contents were also detected parallel with almost the same JA level in the three genotypes. All the PA treatments decreased the JA content, with the highest decrement in SPM-treated plants, but regardless of the genotypes. Exogenous methyl jasmonate has also been reported to increase the production of conjugated PAs in barley [33]. In addition, genes encoding enzymes for synthesis of PAs, such as ADC1 and SPDS1 have been proved to be affected by JA signalling in *Arabidopsis* [57], and exogenous JA induced PAO in rice [34], and regulated the expression of chickpea *CuAO* [58]. However, there are only a few studies about the effects of PAs on the synthesis of JA. PA treatments, especially SPM elicited the biosynthesis of JA in lima bean [35], while constitute overexpression of *SPMS* in *Arabidopsis* increased the levels of expression of genes involved in JA synthesis and signalling [17,59]. Under the present conditions, it was found that all the PAs induced the expression of *AOS*, with the highest level in SPM-treated plants, especially in the SPM-treated *sid2* mutant. The *Arabidopsis AOS* promoter was found to be activated by a variety of signals, including JA, wounding, and even exogenous SA [60], however, SA-induced repression of the JA-signalling pathway is independent of JA biosynthesis, as occurs downstream of JA perception [61]. Despite this, in *eds4* mutant *Arabidopsis* (also with reduced SA content) relieved inhibition of JA-dependent signalling responses were detected, confirming that the SA signalling and JA signalling can be mutually inhibitory [62].

*ADC2*-overexpressing transgenic plants exhibited a reduction in both the contents of GA_1, 4, 9_ contents, and in the expression levels of the *GA20ox1*, *GA3ox1* and *GA3ox3* transcripts [63], suggesting that PUT accumulation represses GA synthesis. SPD treatment decreased GA_3_ content, and *GA3ox* expression in apple terminal buds during floral induction [4]. However, on the other hand, an increased content of GAs was observed in PA-treated plants under drought conditions in creeping bentgrass [64]. SPD increased GA_3_ content in maize [65], and in sweet corn seed embryos [66]. Nevertheless, accumulation or deficiency of PUT, SPD or SPM did not influence the expression of GA signalling gene in transgenic *Arabidopsis* leaves or tomato fruits [67]. These data indicate that the effect of PAs on GA synthesis is highly dependent on plant species and developmental stage. Under the present conditions, PAs treatments did not influence the GA_1_ content, except for a slight decrease in SPM-treated wild type. Although, the *GA3ox1* expression increased after PUT treatment, in each of the genotypes, especially in *sid2* mutant, and after SPD treatment in the mutants, with still a higher extent in case of *sid2*, a dramatic decrease was observed after SPM treatment in all the genotypes. According to these, no negative correlation was found between the accumulation of PUT and the level of GA_1_, while the inhibitory effect of SPM treatment on *GA3ox1* was pronounced in all genotypes. The existence of crosstalk between GAs and SA signalling in *Arabidopsis* was also suggested, as GA treatment increased the endogenous levels of SA and the expression of the *ICS1* gene in Col-0, and the transcript levels of the *GA3ox1* gene were greatly elevated by SA treatments [68,69]. However, under the present conditions, in each cases GA_1_ accumulation was higher in *sid2* mutant than in the wild type, indicating that SA also influences to some extent GA levels in plants. Nevertheless, here, the GA-ABA antagonism should be also taken into consideration, which has been reported during seed development, plant growth and stress responses. Stress-induced increases of ABA level were parallel with decreases in the GA level and the suppression of the GA synthetic enzyme genes in *Arabidopsis* [70,71].

## 4. Materials and Methods

### 4.1. Plant Materials, Growth Conditions and Treatments

In our experiment two *Arabidopsis* SA-deficient mutants were investigated, *eds5-1* (*eds5*), which is a SA transport mutant [44,72] and *sid2-2* (*sid2*), which is a SA biosynthesis mutant [73]. *Arabidopsis* Col-0 was used as control, wild type. Seeds of mutants were obtained through the European *Arabidopsis* Stock Centre (NASC, Sutton Bonington Campus, Loughborough, LE125RD, United Kingdom). The plants were self-pollinated for two generations and the presence of mutation was revealed by genotyping. In the case of *eds5-1* the full length coding sequence was amplified (*EDS* FL_F 5′-ATGCTAATCAAATCCCAAAGA-3′ and *EDS* FL_R 5′-TTTAA TCTTCTCCACCGTGTAT-3′) and a deletion of eight bp was proved by sequencing resulting in a frameshift error. Genotyping of *sid2-2* was carried out with *sid2-2* F (5′-acagcaggataattacggatacc-3′) and *sid2-2* R (5′-ccactctgaagatgggtcact-3) primers [74].

Plants were cultivated hydroponically using an Araponics system (Araponics, Liège, Belgium). Cultures were grown in a 25% modified Hoagland-solution [75] in a Conviron GB-48 plant growth chamber (Controlled Environments, Winnipeg, MB, Canada) under control conditions at 22 °C/20 °C with 8/16 h light/dark period and 75% humidity for 28 days. The photosynthetic photon flux density (PPFD) was 100 µmol m^−2^ s^−2^. The 28-day-old plants were treated with nutrition solution containing 0.5 mM PUT, SPD or SPM. After a one-day exposure of different PA treatments, fully developed leaves and roots were collected. Leaves were frozen immediately in liquid nitrogen, while the roots were washed in distilled water before freezing. Samples were stored at −80 °C until further analysis.

### 4.2. Chlorophyll-A Fluorescence Induction Measurements

The chlorophyll-*a* fluorescence was measured by using a pulse amplitude modulated fluorometer (Imaging-PAM M-Series fluorometer; Walz, Effeltrich, Germany). The maximum quantum yield of PSII photochemistry Fv/Fm was measured on 20 min dark-adapted leaves. Fv/Fm= (Fm-F0)/Fm, where Fm is the maximum fluorescence induced by a saturating flash (8000 μmol m^−2^ s^−1^ PPFD for 0.8s) in dark adapted leaves, F0 is the minimum chlorophyll fluorescence yield in the dark (PPFD < 1 μmol m^−2^ s^−1^). The effective PSII quantum yield (YII) was measured at a light intensity of 250 μmol m^−2^ s^−1^ and represents the proportion of absorbed light energy being used in photochemistry. It is calculated as: (Fm’−F)/Fm’, Fm’ is the maximum fluorescence level induced by a saturating light pulse at the steady state, and F is the steady state chlorophyll fluorescence immediately prior to the flash.

### 4.3. Polyamine Analysis

Samples preparation and pre-column derivatisation with dansyl chloride and HPLC analyses were performed according to Némethetal [19]. The most abundant polyamines, namely PUT, spermidine (SPD) and spermine (SPM) were analysed by HPLC using a W2690 separation module (Waters, Milford, MA, USA) equipped with a 100 × 2.1 mm Kinetex reverse phase column 5 μm (C18) (Phenomenex, Inc., Torrance, CA, USA) and a W474 scanning fluorescence detector with excitation at 340 nm and emission at 515 nm.

### 4.4. Benzoic Acid, Jasmonic Acid, Gibberellin 1, Salicylic Acid and Abscisic Acid Extraction and Analytical Procedure

The sample extraction was done using methanol:water (2:1 *v*/*v* %), 100 mg FW/mL final sample ratio. Ultra-performance liquid chromatography with tandem mass spectrometry (Waters Acquity I class UPLC system coupled to a Waters Xevo TQ-XS instrument equipped with a UniSpray (US) ion source operated in timed MRM mode) analyses were carried out according to Vrhovsek et al. [76] with slight modifications as described in detail by Pál et al. [32]. Separation was achieved on a Waters Acquity HSS T3 column (1.8 μm, 100 mm × 2.1 mm), kept at 40 °C. Mobile phases both contained 0.1 *v*/*v* % formic acid while a water and acetonitrile gradient was used. For quantitation the transition exhibiting the highest S/N ratio was utilized (Table S1). Data processing was performed using Waters MassLynx 4.2 and TargetLynx softwares.

### 4.5. Gene Expression Analysis

Total RNA was extracted from fully developed leaves using TRI Reagent^®^. The samples were treated with DNase I and cleaned with a Direct-zol™ RNA MiniPrep Kit (Zymo Research, Irvine, CA, USA) according to the manufacturer’s instructions. The quality and integrity of RNA was monitored using agarose gel and the samples were quantified with a Nanodrop 2000 spectrophotometer (Thermo Fisher Scientific Inc., Wilmington, MA, USA). Total RNA (1000 ng) was reverse transcribed by using M-MLV Reverse Transcriptase (Promega Corporation, Madison, WI, USA) and oligo(dT)18 (Thermo Fisher Scientific) 1 µL of 2-fold diluted cDNA, gene-specific primers and housekeeping primers (Table S1), PCRBIO SyGreen Mix (PCR Biosystems, London, UK) and CFX96 Touch™ Real-Time PCR Detection System (Bio-Rad, Hercules, CA, USA) were used for quantitative real-time PCR reaction. Melt curve analysis was also performed to confirm the presence of a single PCR product. The relative gene expression values were determined with the 2^−ΔΔCt^ method [77]. Ct values were normalized by the Ct values of housekeeping gene *Atactin8* [78]. All reactions were performed in triplicate using three biological and three technical repetitions.

### 4.6. Statistical Analysis

Three independent biological experiments were performed, and representative data are presented. The results are the means of at least five replicates for spectrophotometric and chromatographic determinations. The data were statistically evaluated using the standard deviation in Microsoft Excel. Different letters indicate statistically significant differences (*p* < 0.05) between multiple groups (one-way ANOVA with Duncan post hoc test was performed using SPSS 16.0. Box-plot presentation for all the investigated parameters in Supplementary Figures S1–S10. (Boxes represent Q1 and Q3 quartiles and the middle line of the box is the median (Q2). Whiskers show the minimum and maximum values.) Analysis of variance of metabolite accumulation and changes in gene expression was performed by using SPSS 16.0, where mean squares (MS) followed by asterisks (*) are significantly different (*p* < 0.05). Supplementary heat map (Supplementary Figure S11), presenting the metabolite accumulation and changes in gene expression, was evaluated using the membership function value (MFV) using the fuzzy comprehensive evaluation method [79]. The MFV was calculated using the following equation: Xi = (X − Xmin)/(Xmax − Xmin) × 100.

## 5. Conclusions

The present study indicates that the individual PAs, namely PUT, SPD and SPM, induced different changes in the investigated *Arabidopsis* plants. Table S1 shows the results of variance analysis in order to detect the effect of genotype, treatment and genotype x treatment interaction. Under control conditions, *sid2* mutant showed a remarkable difference in PA metabolism compared to the other two genotypes only regarding *SPDS2* expression, but this higher transcript level was not accompanied by a higher SPD level. The exogenous PA treatments upregulated the PA metabolism, as de novo synthesis of PUT and SPM is induced, while that of SPD is inhibited, and in parallel with these events, the induction of the terminal catabolism instead of back-conversion is responsible for the unchanged level of higher PAs. The SA deficient *sid2* mutant showed pronouncedly different responses to the individual PA treatments compared to the Col-0 wild type. Pronounced differences were observed in the SA content between the two mutant and the wild type. Although the PA treatments could hardly influence the SA levels, the initial differences still remained. However, the *ICS1* and *PAL1* expression showed PA treatment and genotype dependent changes, suggesting that the induction of the PAL pathway is more predominant upon PA treatment, with the highest upregulation in *sid2* mutant. Remarkable changes in hormone synthesis were also found after PA treatments. Interestingly PUT treatment increased the *GA3ox1* expression, SPD treatment has spectacular effect on ABA content and *NCED* expression, while SPM application influenced rather JA content and *AOS* expression. In several cases the most pronounced difference in the hormone biosynthesis after PA treatments were found in *sid2* mutant compared to the Col-0 wild type (Figure S11). However, these differences at transcript levels were not always in accordance with the hormone contents, suggesting, that synergetic or antagonistic interactions between these plant hormones should also be taken into consideration. Thus, understanding the link between PA metabolism/signalling and plant hormone signalling needs further studies from this point of view, using plant hormone synthesis mutants.

## Supplementary Materials

Supplementary materials can be found at https://www.mdpi.com/1422-0067/20/22/5746/s1.

## Abbreviations

ABA	abscisic acid
ADC	arginine decarboxylase
AOS	allene oxide synthase
CuAO	cooper amine-oxidase
CS	chorismate synthase
GAs	gibberellins
GA3ox	gibberellin 3-oxidase
ICS	isochorismate synthase
JA	jasmonic acid
ADC	arginine decarboxylase
NCED	9-*cis*-epoxycarotenoid dioxygenase
PAL	phenylalanine ammonia-lyase
PAO	polyamine oxidase
PUT	putrescine
SA	salicylic acid
SPD	spermidine
SPDS	spermidine synthase
SPM	spermine
SPMS	spermine synthase
