# Supplementary material for: Polyamine-Induced Hormonal Changes in eds5 and sid2 Mutant Arabidopsis Plants
Source: Int J Mol Sci. 2019 Nov 15;20(22):5746. doi: 10.3390/ijms20225746 (PMC6887987; doi:10.3390/ijms20225746)
Supplement: Supplementary file 1 [file ijms-20-05746-s001.pdf]

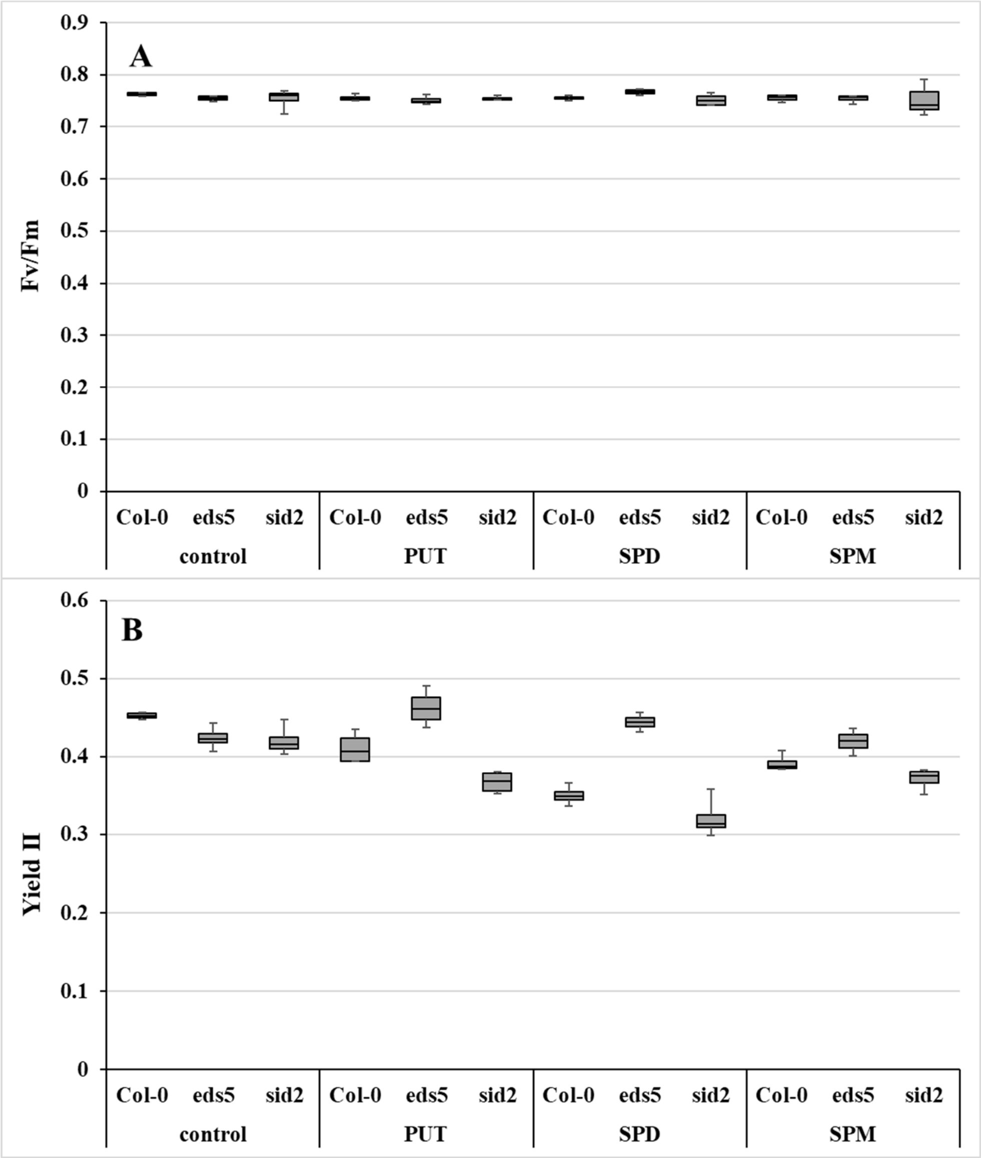

Figure S1. Effect of 0.5 mM 1-day of putrescine (PUT), spermidine (SPD) and spermine (SPM) treatments on the chlorophyll-*a* fluorescence induction parameters (A: Fv/Fm: maximum quantum yield of PSII photochemistry, B: YII: effective PSII quantum yield) in Col-0, wild type, *eds5-1* (*eds5*) and *sid2-2* (*sid2*) *Arabidopsis* mutants. Data represent mean values ± SD. Different letters indicate significant differences at P≤0.05 level.

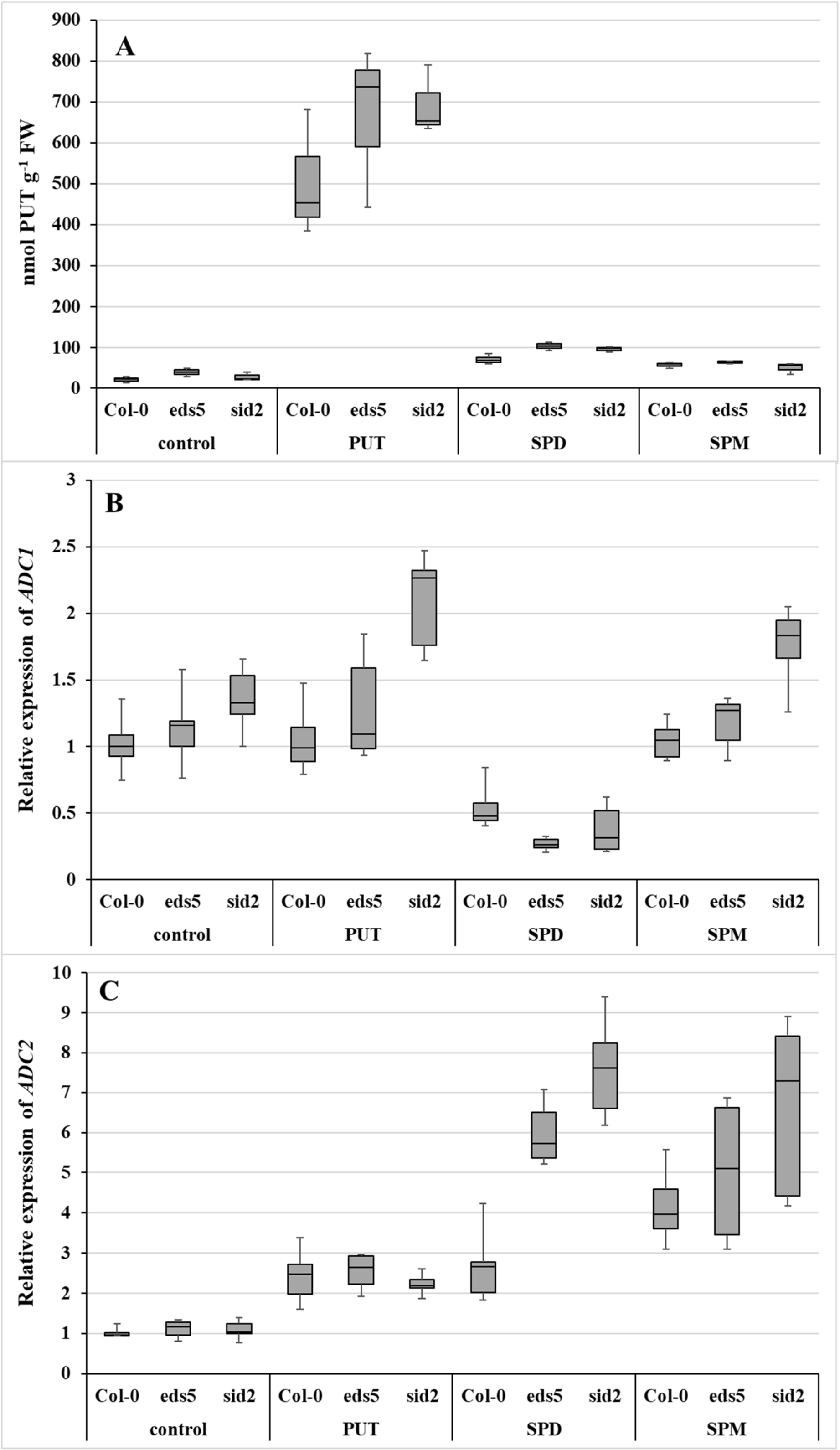

Figure S2. Effect of 0.5 mM 1-day of putrescine (PUT), spermidine (SPD) and spermine (SPM) treatments on the endogenous putrescine content (A: PUT) and on the expression levels of putrescine synthesis genes (B-C: ADC1-2: arginine decarboxylase1-2) in Col-0, wild type, eds5-1 (eds5) and sid2-2 (sid2) Arabidopsis mutants. Data represent mean values ± SD. Different letters indicate significant differences at P≤0.05 level.

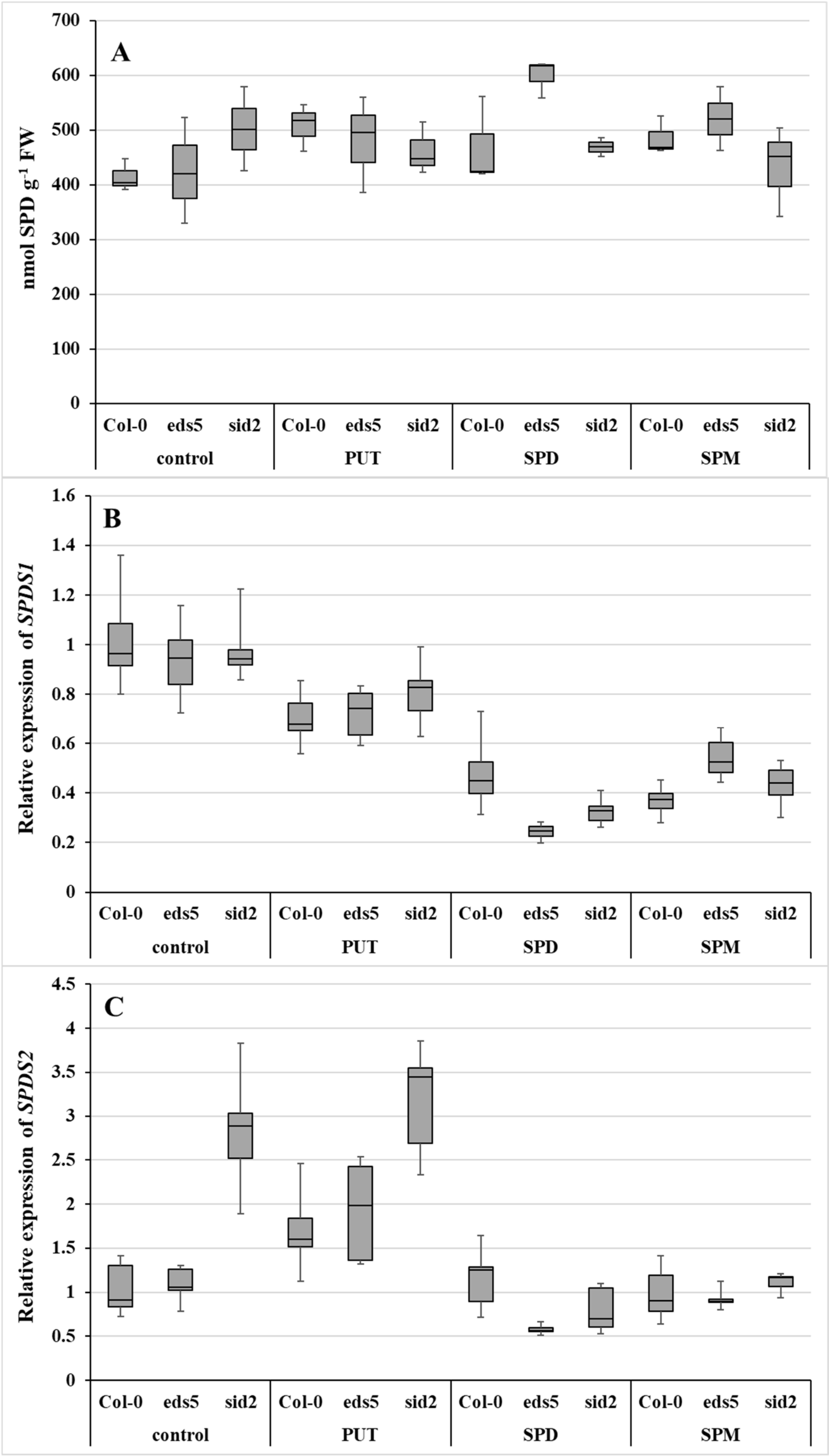

Figure S3. Effect of 0.5 mM 1-day of putrescine (PUT), spermidine (SPD) and spermine (SPM) treatments on spermidine content (A: SPD) and on the expression levels of spermidine synthesis genes (B-C: *SPDS1-2*: spermidine synthase1-2) in Col-0, wild type, *eds5-1* (*eds5*) and *sid2-2* (*sid2*) *Arabidopsis* mutants. Data represent mean values ± SD. Different letters indicate significant differences at P≤0.05 level.

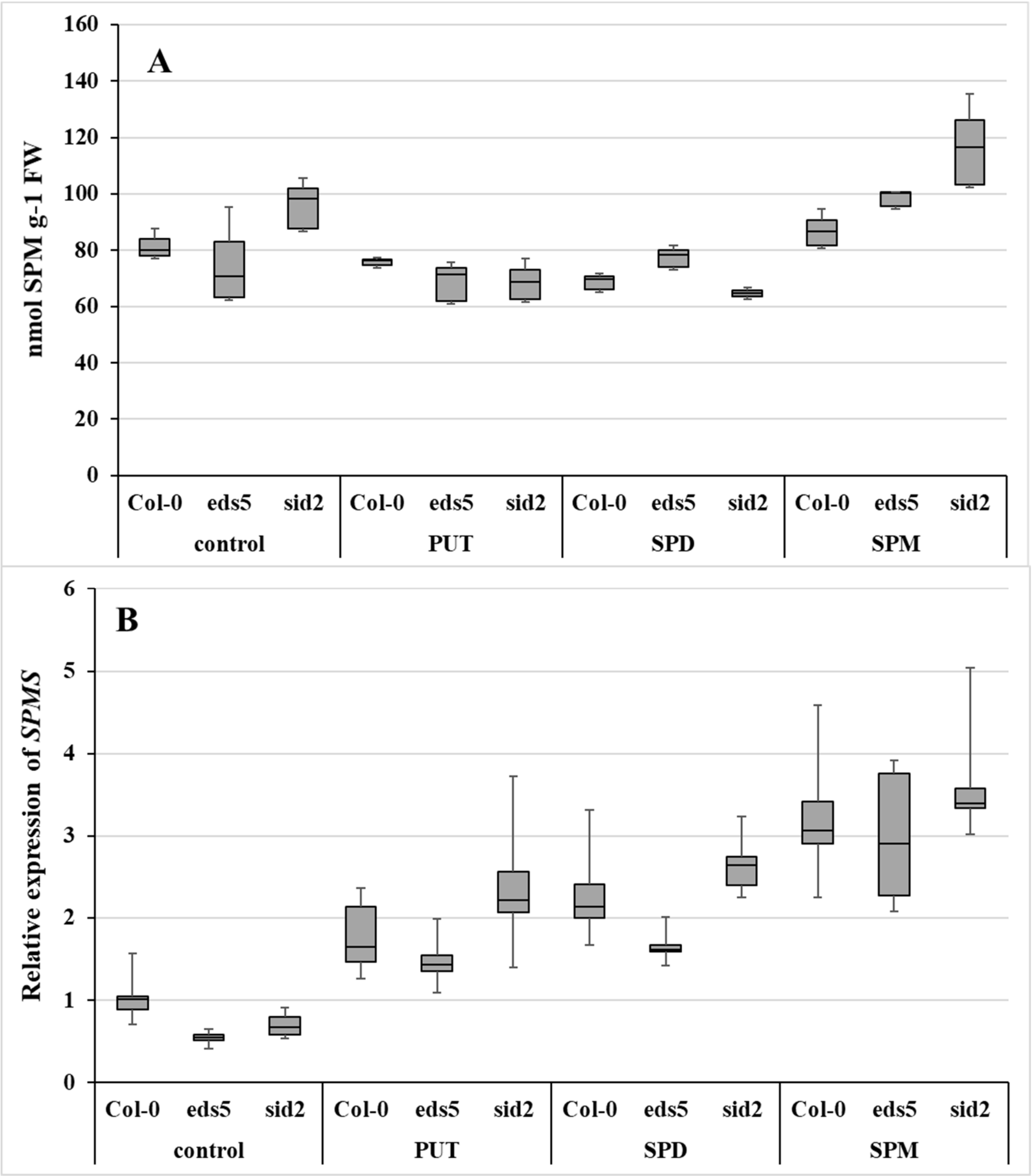

Figure S4. Effect of 0.5 mM 1-day of putrescine (PUT), spermidine (SPD) and spermine (SPM) treatments on spermine content (A: SPM) and on the expression levels of spermine synthase gene (B: *SPMS*) in Col-0, wild type, *eds5-1* (*eds5*) and *sid2-2* (*sid2*) *Arabidopsis* mutants. Data represent mean values ± SD. Different letters indicate significant differences at P≤0.05 level.

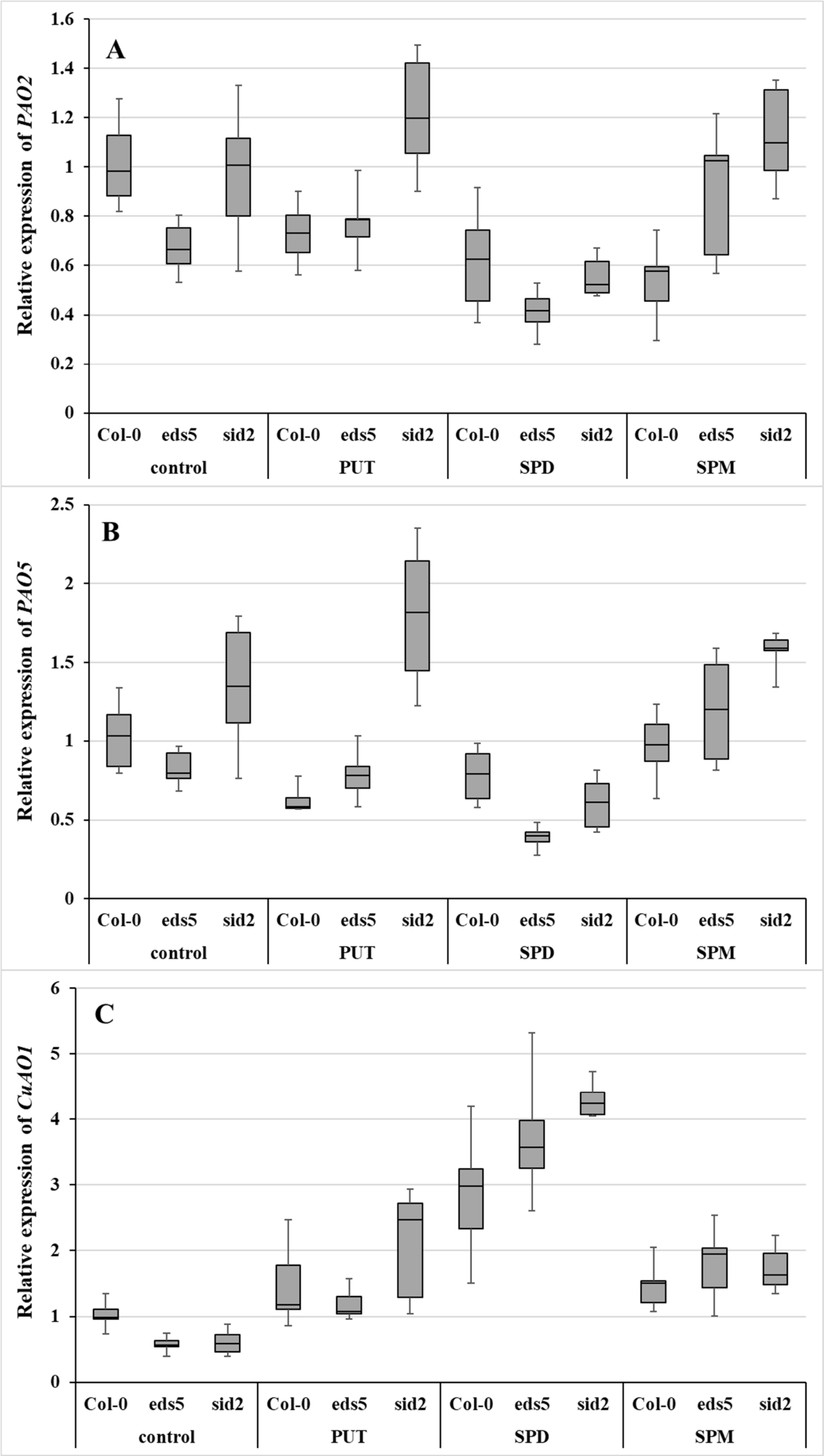

Figure S5. Effect of 0.5 mM 1-day of putrescine (PUT), spermidine (SPD) and spermine (SPM) treatments on the expression levels of polyamine metabolism genes (A-B: *PAO2-5*: polyamine oxidase2-5, C: *CuAO1*: cooper amine-oxidase1) in Col-0, wild type, *eds5-1* (*eds5*) and *sid2-2* (*sid2*) *Arabidopsis* mutants. Data represent mean values ± SD. Different letters indicate significant differences at P≤0.05 level.

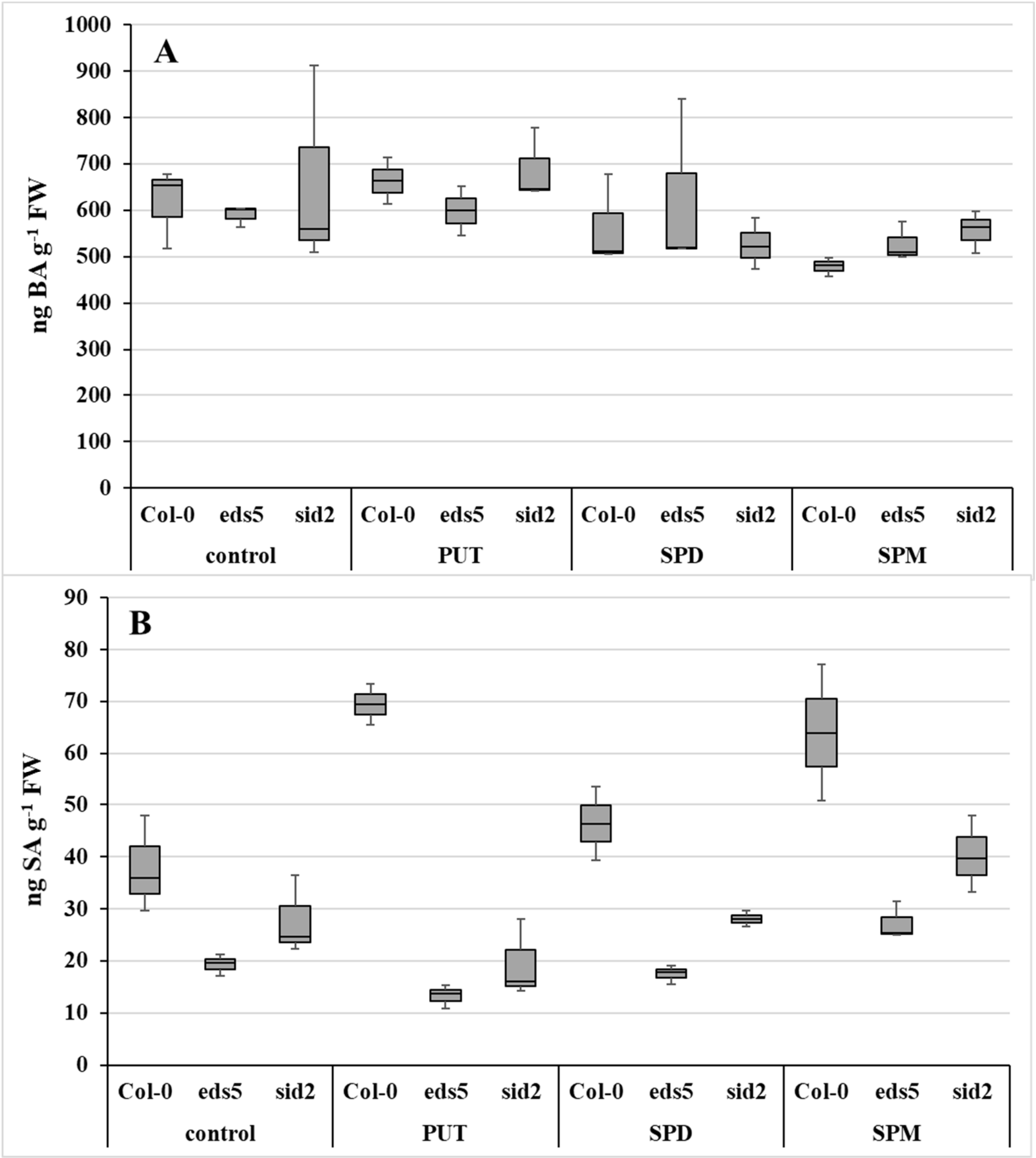

Figure S6. Effect of 0.5 mM 1-day of putrescine (PUT), spermidine (SPD) and spermine (SPM) treatments on the contents of benzoic acid (A: BA) and salicylic acid (B: SA) in Col-0, wild type, *eds5-1* (*eds5*) and *sid2-2* (*sid2*) *Arabidopsis* mutants. Data represent mean values ± SD. Different letters indicate significant differences at P≤0.05 level.

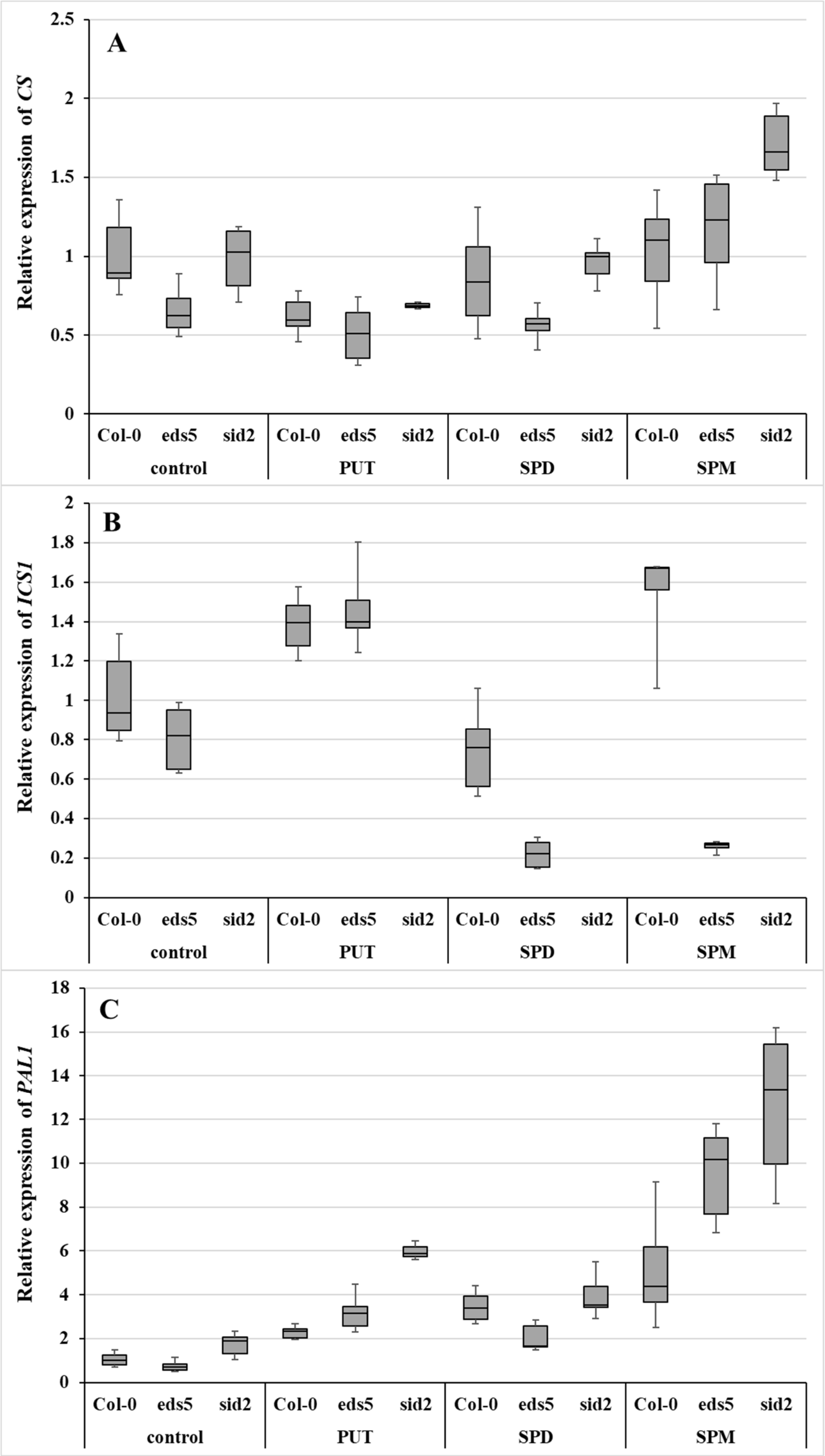

Figure S7. Effect of 0.5 mM 1-day of putrescine (PUT), spermidine (SPD) and spermine (SPM) treatments on the expression levels of genes involved in salicylic acid synthesis (A: *CS*: chorizmate synthase, B: *ICS1*: isochorismate synhtase1, C: *PAL1*: phenylalanine ammonia-lyase1) in Col-0, wild type, *eds5-1* (*eds5*) and *sid2-2* (*sid2*) *Arabidopsis* mutants. Data represent mean values ± SD. Different letters indicate significant differences at P≤0.05 level.

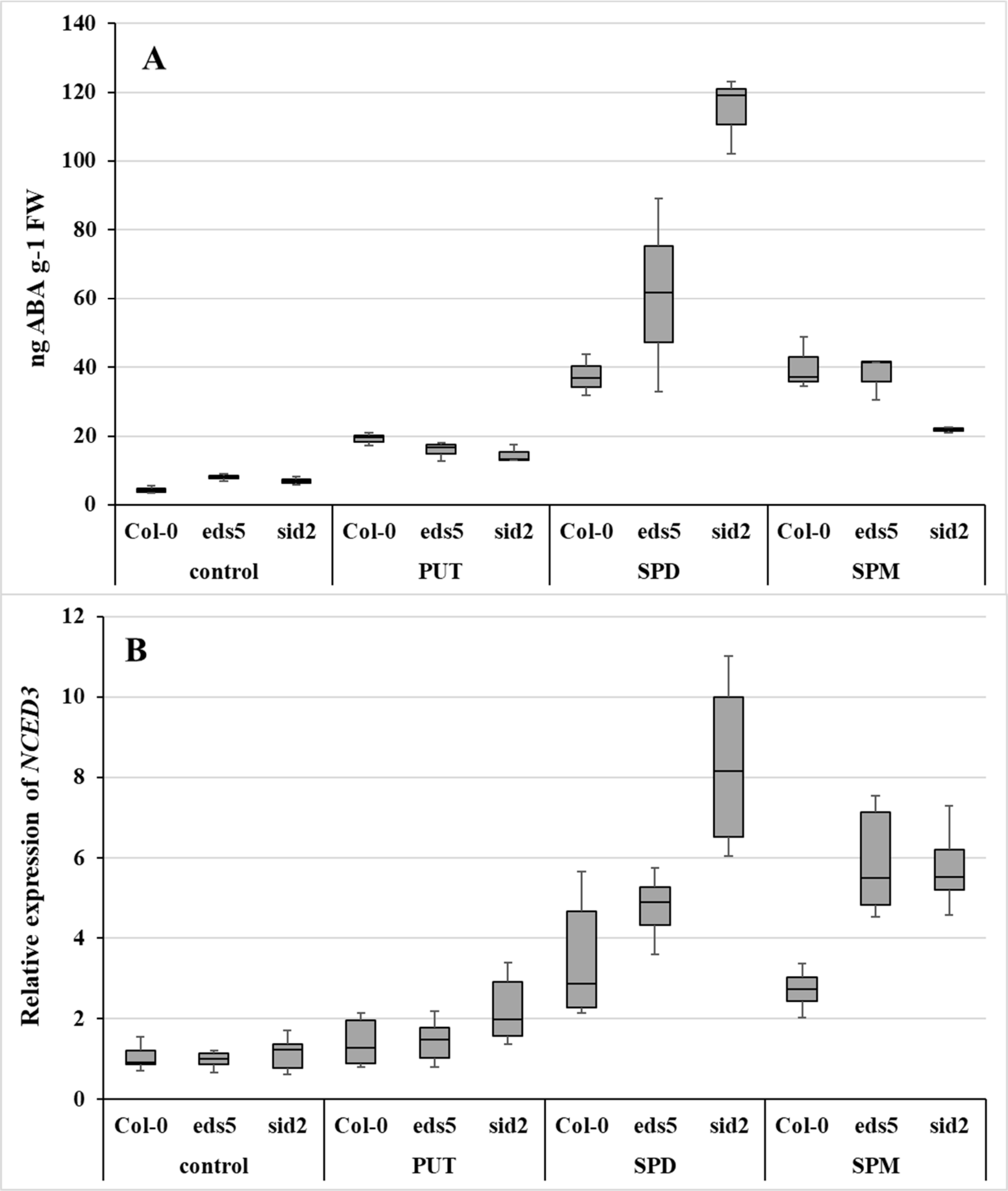

Figure S8. Effect of 0.5 mM 1-day of putrescine (PUT), spermidine (SPD) and spermine (SPM) treatments on the content of abscisic acid (A: ABA) and the expression level of gene involved in abscisic acid synthesis (B: *NCED3:* 9-cis-epoxycarotenoid dioxygenase3) in Col-0, wild type, *eds5-1* (*eds5*) and *sid2-2* (*sid2*) *Arabidopsis* mutants. Data represent mean values ± SD. Different letters indicate significant differences at P≤0.05 level.

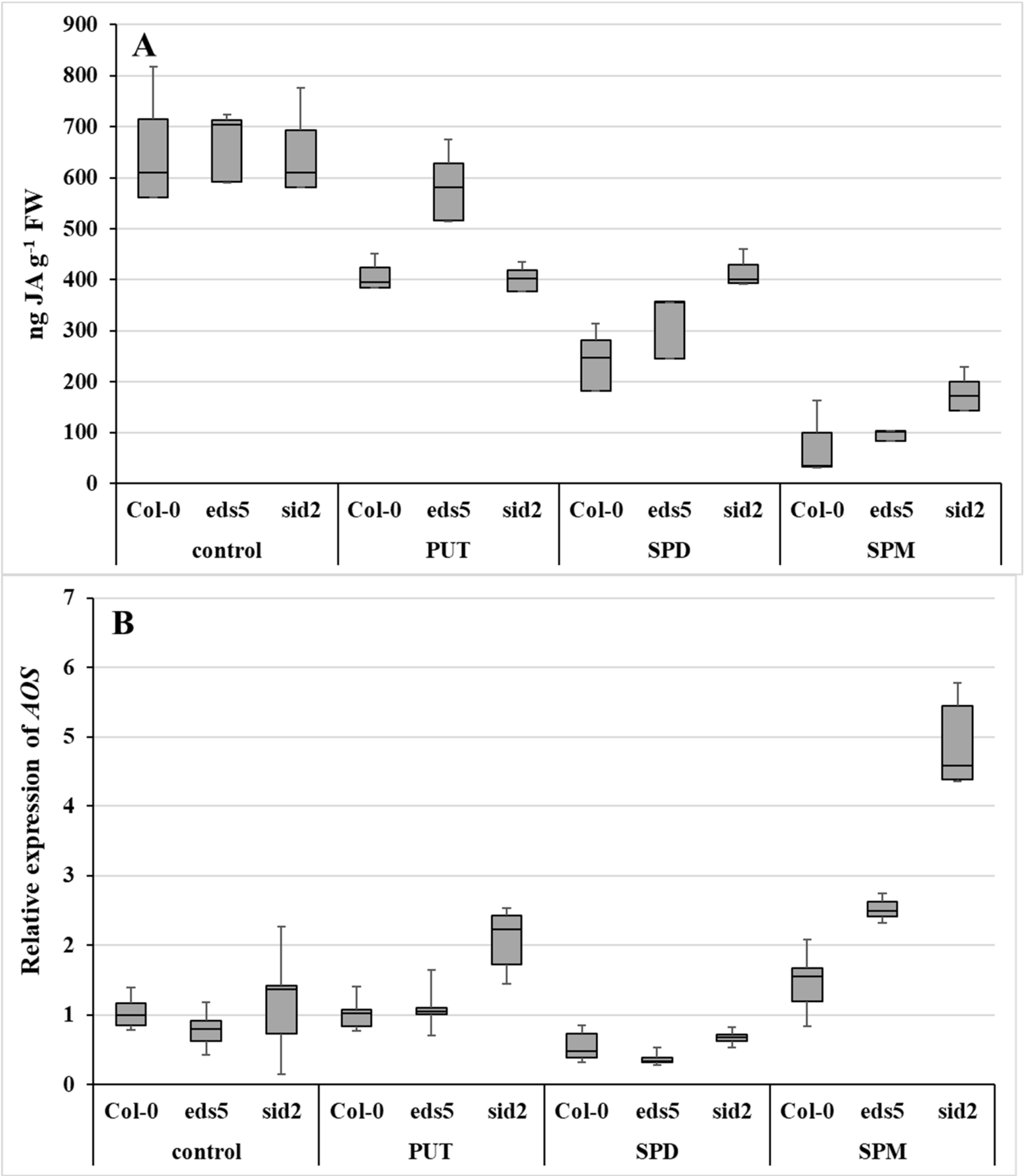

Figure S9. Effect of 0.5 mM 1-day of putrescine (PUT), spermidine (SPD) and spermine (SPM) treatments on the content of jasmonic acid (A: JA) and the expression level of gene involved in jasmonic acid synthesis (B: *AOS:* allene oxide synthase) in Col-0, wild type, *eds5-1* (*eds5*) and *sid2-2* (*sid2*) *Arabidopsis* mutants. Data represent mean values ± SD. Different letters indicate significant differences at P≤0.05 level.

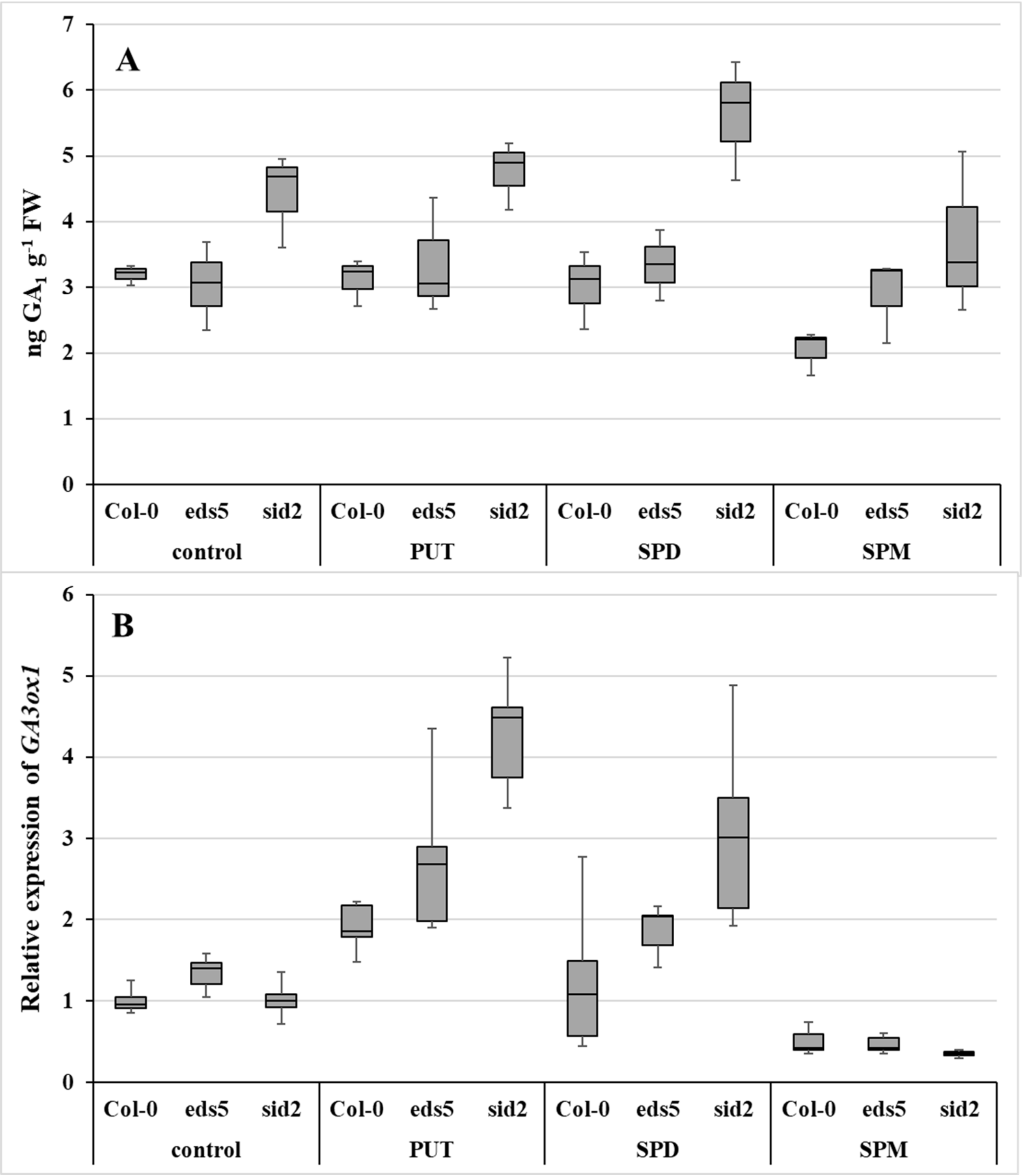

Figure S10. Effect of 0.5 mM 1-day of putrescine (PUT), spermidine (SPD) and spermine (SPM) treatments on the content of gibberellic acid (A: GA1) and the expression level of gene involved in gibberellic acid (B: *GA3ox1:* gibberellin 3-oxidase) in Col-0, wild type, *eds5-1* (*eds5*) and *sid2-2* (*sid2*) *Arabidopsis* mutants. Data represent mean values ± SD. Different letters indicate significant differences at P≤0.05 level.

Table S1. Factorial analysis of variance (SPSS 16.0) for determination of the effect of genotypes (G), polyamine treatments (T) and G×T.

Sourse of variation	df	MS																					
		PUT	*ADC1*	*ADC2*	SPD	*SPDS1*	*SPDS2*	SPM	*SPMS*	*PAO2*	*PAO5*	*CuAO1*	BA	SA	*CS*	*ICS1*	*PAL1*	ABA	*NCED3*	JA	*AOS*	GA_1	*GA3ox1*
Genotype (G)	2	3 374 406.33	**2.43***	**30.41***	1 247 731.86	0.01	**8.17***	74 102.78	**3.62***	**0.78 ***	**2.92***	**3.30***	2 503.86	**3203.98***	**0.78***	**11.06***	**68.19***	**617.61***	**42.52***	15 288.52	**13.14***	**10.83***	**2.11***
Treatment (T)	3	**30185247.36***	**6.30***	**120.09***	1 361 486.47	**2.2***	**11.61***	**713813.13 ***	**25.92***	**0.85***	**2.10***	**33.39***	29 064.10	**369.96***	**1.73***	**1.50***	**312.19***	**7262.55***	**132.31***	**423212.88***	**18.80***	**2.04***	**12.43***
GxT	6	2 532 490.00	**0.64***	**13.32***	1 794 605.97	**0.07***	**2.74***	123 484.78	**0.51***	**0.35***	**0.73***	**1.61***	6 611.27	**230.46***	**0.21***	**0.80***	**25.75***	**1468.21***	**14.57***	15 851.56	**4.30***	0.45	**2.90***

*: significant at P≤0.05 level.

R Squared = PUT (0.643), *ADC1* (0.852), *ADC2* (0.871), SPD (0.465), *SPDS1* (0.87), *SPDS2* (0.859), SPM (0.693), *SPMS* (0.825), *PAO2* (0.7), *PAO5* (0.785), *CuAO1* (0.852), BA (0.357), SA (0.889), *CS* (0.738), *ICS* (0.951), *PAL1* (0.895), ABA (0.937), *NCED3* (0.875), JA (0.869), *AOS* (0.908), GA_1 (0.727), *GA3ox1* (0.792)

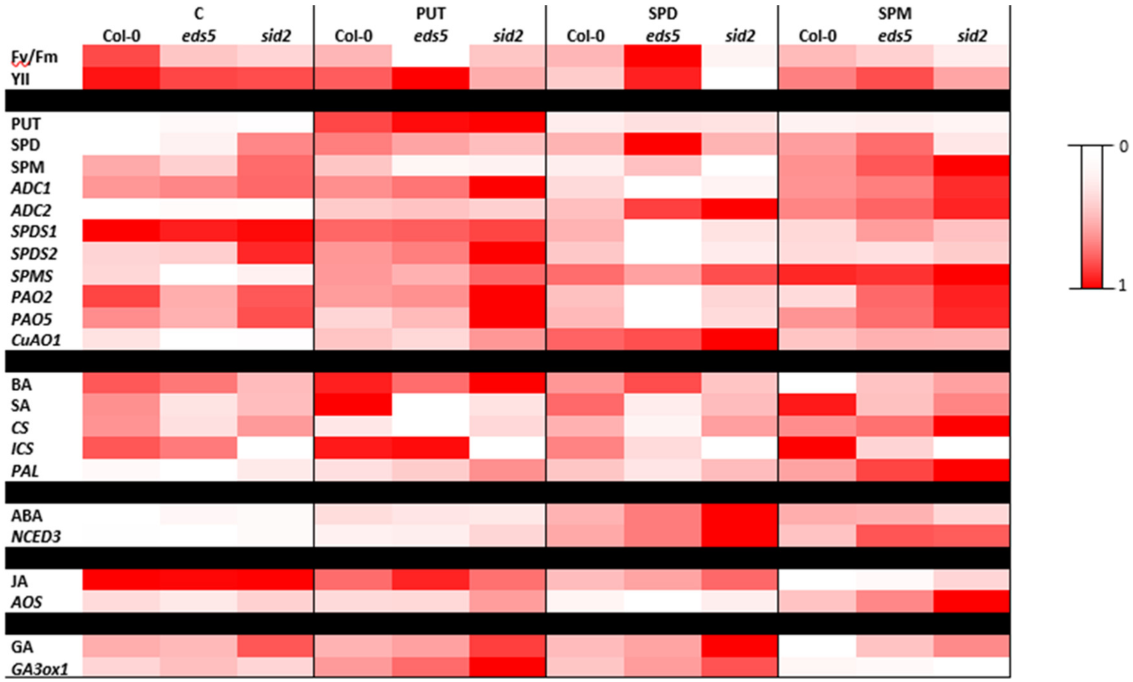

Figure S11. Heat map presenting the metabolite accumulation and changes in gene expression, was evaluated using the membership function value (MFV) using the fuzzy comprehensive evaluation method (Chen et al., 2012). The MFV was calculated using the following equation: $X_i = (X - X_{min})/(X_{max} - X_{min}) \times 100$.
